# Performance measures across playing categories in competitive golfers with disabilities: a cross-sectional study

**DOI:** 10.3389/fspor.2026.1748973

**Published:** 2026-05-07

**Authors:** Kristian M. Jones, William Wynter Bee, Brad Stenner, Roger Hawkes, Eric S. Wallace

**Affiliations:** 1 Equipment Standards, The R&A, St Andrews, United Kingdom; 2 Institute for Sport, PE and Health Sciences, Moray House School of Education and Sport, University of Edinburgh, Edinburgh, United Kingdom; 3 EDGA, Wassenaar, Netherlands; 4 Alliance for Research in Exercise, Nutrition and Activity (ARENA), Allied Health and Human Performance, Adelaide University, Adelaide, SA, Australia; 5 School of Sport and Exercise Science, Ulster University, Belfast, United Kingdom

**Keywords:** adaptive sport performance, golf performance, golfers with disabilities, launch monitor, paralympic

## Abstract

**Objectives:**

The aims of the study were to establish normative full golf swing launch data for golfers with disabilities and to examine the relationships between Sport Class and swing performance.

**Methods:**

142 competitive golfers (mean ± SD, age = 40.6 ± 14.1 years, 27 female, 115 male), across four disability Classes struck shots with three different clubs. Club and ball launch variables were measured using a Doppler-radar based launch monitor. Descriptive statistics and generalized linear models were used to establish normative data and within-class effects.

**Results:**

These novel normative data across seven key performance variables indicate a wide range of ball-striking ability and consistency both between and within classes. The Sitting playing style displayed lower carry distance [*B* = −60.9, SE = 7.3, *p* < 0.001, 95% CI (−75.2 −46.6), partial *f*^2^ = 0.17], higher carry distance variability [*B* = 2.4, SE = 1.0, *p* = 0.013, 95% CI (0.5 4.3), partial *f*^2^ = 0.01] and lower variability in offline distance [*B* = −2.9, SE = 0.7, *p* < 0.001, 95% CI (−4.4 −1.5), partial *f*^2^ = 0.04]. The visual playing style displayed higher carry distance variability [*B* = 2.6, SE = 0.8, *p* = 0.002, 95% CI (1.0 4.2), partial *f*^2^ = 0.02]. No other statistical performance differences were observed.

**Conclusion:**

The golfers displayed a wide range of ability in the key performance variables studied. Sitting golfers displayed significantly lower carry distance with all clubs compared to the other categories. These normative data will inform future research, governing bodies and tournament organizers.

## Introduction

Normative data (norms) comprise observations from a reference population which characterize what is usual or expected in a defined population. Norms may be readily obtained from cross-sectional studies. Such data, which seek to describe rather than explain phenomena, are essential in the branch of medical science that deals with the classification of diseases (1). Without established norms for a population, interpreting individual findings can be a significant challenge. Norms are also fundamental for hypothesis generation, as observations which deviate from established norms can indicate areas of further research interest.

Classification is an integral part of disability sport, aiming to minimize the effect of impairment on outcomes and ensuring that sporting excellence, not degree of impairment, determines success (2). The International Paralympic Committee (IPC) Code mandates that evidence-based classification systems are used to unambiguously classify athletes by degree of impairment for each specific sport (3). Golf performance requires a multi-faceted array of skills (both cognitive and physical), which complicates the development and evidencing of sport-wide classification systems.

EDGA (formerly the European Disabled Golf Association) is a not-for-profit, volunteer organization, active in the promotion and delivery of opportunities to sample, participate and compete in golf. EDGA Sport Classes, which were first introduced at the 2023 G4D Open (4), include two categories for intellectual impairment (Intellectual 1 & Intellectual 2), three categories for golfers with limb, balance, range of motion or strength impairments (Standing 1, Standing 2 & Standing 3), two categories for seated golfers (Seated 1 & Seated 2), and two categories for visual impairment (Visual 1 & Visual 2). In all categories, a lower number indicates a greater impairment; for instance, Visual 1 includes golfers who are totally blind, whilst Visual 2 includes golfers with a significant visual impairment (see Supplementary Material S1 for full description of EDGA Sport Classes).

To be successful in golf, a player must be able to consistently execute a wide range of shot types on demand (for example driving, approach shots, chipping and putting), while also possessing the required psychological and strategic skills to manage their performance over the duration of a round. Successful shots in golf require consistent distance (sometimes maximal) and accuracy control to hit the ball as close as possible to the intended target. Several studies have shown that physical factors, including strength (5, 6) and range of motion (6), psychological factors (7), and visual factors (8) have an impact on golf performance. However, whilst there are well-established normative data for golfers without disability, there are no published data reporting these variables for golfers with disabilities.

According to the IPC Code (3), sport-specific classification systems rely on research which focuses on the relationships between impairment and key performance determinants in the sport. The EDGA Sport Classes are based on impairment assessments of the player, performed by accredited assessors. While the Classes have a sound theoretical basis, there is no known research which links performance to classification for competitive golfers with disabilities.

The position of the IPC (2) is to favor research which focuses on the taxonomic principles underpinning classification systems (Process-focused research) rather than research which examines relationships within and between existing classifications (Product-focused research). Whilst process-focused research is foundational to the development of classification systems, there are several instances where product-focused research has provided actionable insight in other paralympic sports. For example, Burkett et al. (9) found that certain categories in para-swimming were unable to satisfactorily differentiate performance and Liljedahl et al. (10) found the same in para-cycling. These findings do not seek to undermine existing categories but instead describe the performance of the competitive population and identify areas where future process-focused research could be undertaken.

Launch data can differentiate between skill levels in able-bodied golfers (11) and can act as a valuable, standardized, sport-specific measure of performance. Therefore, the aim of the present study was to determine normative full golf swing launch data for competitive golfers with disabilities, and to examine the relationships between Sport Classes and swing performance.

## Materials and methods

### Test locations

This cross-sectional study involved a single visit for golfers with disabilities to one of eight golf driving ranges associated with tournaments in which they were competing. Data were pooled from the test sites: the 2023 Amendoeira Open, the 2023 G4D Open (Golf for Disabled/ G4D Tour), the 2023 US Adaptive Open, the 2023 ISPS HANDA World Invitational (G4D Tour), the 2023 South Australian Inclusive Championship, the 2023 Vic Open All Abilities, the 2023 Queensland All Abilities Open, the 2024 G4D Open (G4D Tour), and the 2024 US Adaptive Open. The G4D Open, US Adaptive Open, and G4D Tour events represent the highest level of competition for golfers with disabilities. The events were chosen to provide a large sample of competitive golfers with disabilities, across a range of competition levels, and across a wide range of disability categories. Although weather differed at each test location, no extreme weather (such as high winds) was encountered during the testing.

### Recruitment

Following ethical approval (REF: FC04 2022-23), a total of 162 golfers were recruited through communication via the respective event organizers. Following initial responses, participants were provided with the Participant Information Sheet and a Health History Questionnaire by email prior to the test session. Further oral information about the study procedures and written informed consent were provided and obtained on their visit to the test site.

### Participants and eligibility criteria

Participants were required to be eligible for competition in the Class to which they were assigned and to be registered to compete in the specific event. They were required to be free from injury at the time of testing.

In events where the EDGA Sport Classes were not used (for example, the US Adaptive Open), each participating golfer's Sport Class was determined by a qualified assessor and an author of the study using information provided about their impairment and historical competition data. As Sport Classes are based on medical diagnosis rather than performance-based tests, this process enabled all participants to be classified with an EDGA competition Class.

A total of 162 golfers undertook the testing. Twenty golfers were excluded from the analysis due to not completing the full testing protocol, due to time constraints, physical constraints or fatigue. The test took place at competitions and, understandably, these participants prioritized their competition performance rather than finishing the test. This resulted in a final sample of 142 golfers (mean ± SD, age = 40.6 ± 14.1 years, 27 female, 115 male). Of these golfers, 23 were single leg amputee golfers (5 lead leg above knee, 8 lead leg below knee, 3 trail leg above knee, and 7 trail leg below knee).

### Data collection

Data collection took place on outdoor driving ranges at the competition events noted above. Prior to data collection, participants completed a self-directed warm up, as they would prior to taking part in normal golf activity. The participants were asked to hit shots with each of the 3 test clubs until 10 “acceptable” shots with each club had been completed. Shot attempts which the participant felt were reasonably representative of their ability and which were recorded successfully were deemed acceptable, whilst shot attempts which were clearly mishits (as identified by the participant) or not recorded by the launch monitor were repeated where possible.

Where applicable, each participant used their own Driver, 6-iron, and pitching wedge clubs as the 3 test clubs. These clubs represented “normal” club selection for maximum shot distance on par-5s and long par-4s, a moderate distance approach shot to a green, and a full approach shot to a green from their pitching wedge distance, respectively. 11 participants did not carry a “6-iron” in their set of clubs. In those instances (111 shots), they instead used the equivalent club that they used for that distance and shot type. This included 10 shots struck with a 5-iron, 37 shots struck with a 6 hybrid, 18 shots struck with a 7 hybrid, 36 shots struck with a 7-iron, and 10 shots struck with an 8 hybrid. The designation of “6-iron” is nominal and does not relate to any physical characteristic of the clubhead, so these shots were included in the analysis as they still represented the desired shot type for those golfers. Clubs were used in the order selected by the participant.

Shots were directed towards a target line on the range, defined by an identifiable landmark or flag downrange. Golfers used modern Tour standard balls provided to them on the test range. Ball type was not consistent between test venues but, based on internal conformance testing data, the difference between the ball types used was minimal compared to the expected differences between groups of golfers. As such, the impact of ball type on performance is expected to be negligible. Participants were asked to hit full shots as they would normally perform on the course (i.e., aiming for consistent shot distance and for the shot to finish as close to the target line as possible).

Shot outcome, ball launch, and clubhead variables were measured using a Doppler-radar based launch monitor (Trackman 4, Vedbæk, Denmark). The launch monitor was positioned according to manufacturer guidelines, −3 m behind the teeing area, and the target line was calibrated within the device software. The variables of interest are shown in Table 1. Throughout this paper, units and terminology have been used which are common across the golf industry, thereby making the findings more accessible to stakeholders and practitioners within the sport.

**Table 1:** Definition of variables of interest measured by the launch monitor (12).

Measure	Unit	Definition
Clubhead Speed	mph	The linear speed of the club head's geometric center just prior to first touch with the golf ball.
Ball Speed	mph	The speed of the golf ball's center of gravity immediately after separation from the club face.
Ball Launch Angle	degrees	The vertical angle the golf ball takes off at relative to the horizon and measured immediately after separation from the club face.
Ball Spin Rate	rpm	The rate of rotation of the golf ball about the imaginary line the golf ball rotates around measured immediately after separation from the club face.
Ball Spin Axis	degrees	The angle relative to the horizon of the imaginary line that the golf ball rotates around and is measured after separation from the club face.
Carry distance	yards	The straight-line distance between where the golf ball was launched from and where the ball flight trajectory crosses the plane with the same elevation.
Offline distance	yards	The perpendicular distance between the target line and point where the ball flight trajectory crosses the plane with the same elevation.

### Data analysis

For each of the 142 golfers, data included at least 6 shots with each of the 3 clubs. It has been shown that 6 shots result in a 0.2 mph 95% confidence interval for the median absolute deviation of clubhead speed, based on a normal distribution of shots and observed variability of amateur golfers (11). The average number of shot attempts per participant was 10.2 ± 1.1, 10.4 ± 1.3, and 10.2 ± 1.2 for driver, 6-iron, and pitching wedge respectively (mean ± standard deviation).

For each participant, the individual median and median absolute deviation of the outcome measures (clubhead speed, ball speed, ball launch angle, ball spin rate, ball spin axis angle, carry distance, and offline distance) were calculated for each club. These were the outcome measures on which subsequent analysis was applied.

Descriptive statistics (Mean and Standard Deviation) for all categories and outcome measures is provided as the basis of normative data. The choice to use median and median absolute deviation at an individual level was to ensure intra-individual data were robust to outliers. Mean and standard deviation were used at a group level. Percentile data (including the median) are included in Supplementary Material S3.

For a medium to large effect size (Cohen's f = 0.35), an *a priori* sample size calculation estimated a required group size of 13 (alpha, *α* = 0.05; power, 1-*β* = 0.8; df = 6). This calculation was performed for a fixed effects ANOVA with main effects using G*Power (v3.1.9.7, HHU). The calculation used the effect size from means procedure and assumed a carry distance of 200 yards, a standard deviation of 20 yards within each group, and a difference of 20 yards between groups. These assumptions were based on anecdotal understanding of the population of interest and reported performance of able-bodied golfers (11).

Due to limited numbers of participants in the Intellectual, Sitting, and Visual categories, only three hypotheses were considered for statistical analysis: (1) Differences between the playing style categories (Standing, Sitting, Intellectual, and Visual), (2) Differences between the Sport Classes within the Standing category (Standing 1, Standing 2, and Standing 3), and (3) Differences between single leg amputee golfers based on the site of their amputation (lead leg above knee, lead leg below knee, trail leg above knee, and trail leg below knee—where lead and trail leg are toward and away from the direction of target ball flight respectively. The lead leg is the left leg for a right-handed golfer).

Due to the correlation between outcome measures, statistical analyses were only performed for the carry distance and offline distance outcome measures. Both performance (i.e., the intra-individual median) and variability (the intra-individual median absolute deviation) were considered leading to a total of 12 comparisons (three hypotheses×two outcome measures×two characteristics).

The assumptions of homogeneity of variances and normality were assessed with Levene's and Kolmogorov–Smirnov tests, respectively. Due to violations of these assumptions, ANOVA was not used to analyze the data. Instead, data were analyzed using a generalized linear model (GLM) with a normal distribution and an identity link function. This GLM was used to investigate the effect of Sport Class and Club on the outcome measures. The reference categories were “Standing”, or “Standing 2”, or “lead below knee” (depending on the hypothesis) and “Driver” for Club. Club was included in the model due to its expected effect; however, the primary focus was to assess the effect of Sport Class, and reporting is centered on this effect.

A Bonferroni correction for multiple comparisons was applied at the GLM level—12 hypotheses were considered and the adjusted alpha significance level was 0.004 (0.05/12). Cohen's *f^2^* and partial *f^2^* were calculated as standardized effect size measures at the model and coefficient level respectively (13). All data analysis was performed in MATLAB (r2023b, Mathworks, Natick, MA).

## Results

**Figure 1:**
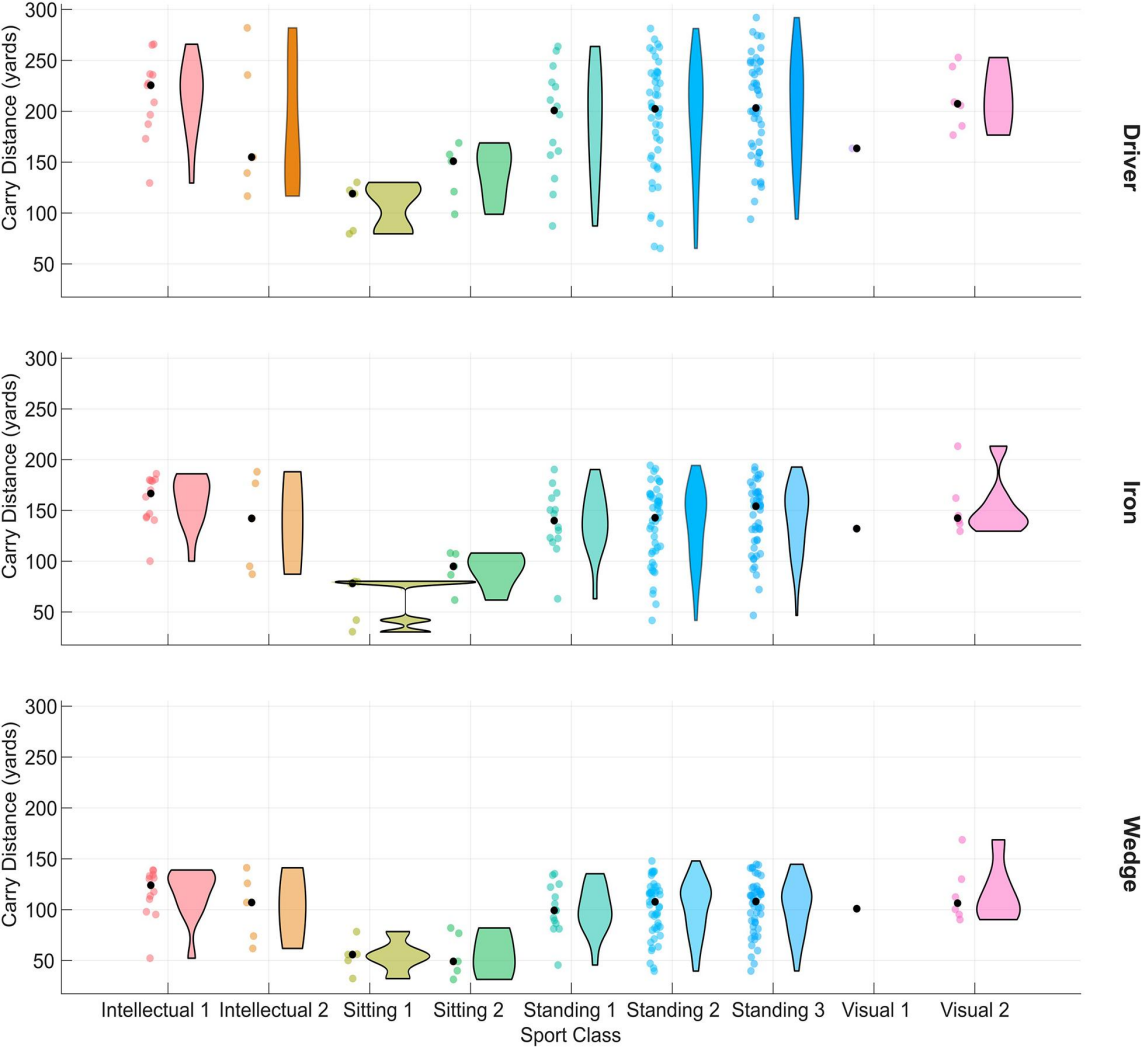
Carry distance by sport class for the three clubs tested. Individual points show golfers median carry distance. Bold points show median of each playing category and violin elements show distributions.

Figure 1 shows representative normative data for carry distance according to Sport Class.

The intra-individual median values for the outcome measures (clubhead speed, ball speed, ball launch angle, ball spin rate, ball spin axis angle, carry distance, and offline distance) for the playing style categories are provided in Table 2. The GLM for carry distance performance was statistically significant (*F* = 102.0, *p* < 0.001, *f^2^* = 1.21). The Sitting playing style was associated with lower carry distance in the GLM [*B* = −60.9, SE = 7.3, *p* < 0.001, 95% CI (−75.2 −46.6), partial *f*^2^ = 0.17]. For offline distance performance, the GLM was not statistically better than a constant model (*F* = 0.664, *p* = 0.651, partial *f^2^* = 0.01).

**Table 2:** Performance (intra-individual median—top) and variability (intra-individual median absolute deviation—bottom) measures for playing style for the three clubs tested.

Club	Sport Class	N	Clubhead Speed (mph)		Ball Speed (mph)		Ball Launch Angle (degrees)		Ball Spin Rate (rpm)		Ball Spin Axis (degrees)		Carry Distance (yards)		Offline Distance (yards)	
			Mean	SD	Mean	SD	Mean	SD	Mean	SD	Mean	SD	Mean	SD	Mean	SD
Driver	Intellectual	17	95.7	16.0	139.0	25.0	11.3	3.3	3,020	644	2.5	11.9	206.2	49.5	−1.5	12.4
	Sitting	10	67.3	8.4	98.8	12.5	13.9	2.3	2,728	633	6.6	13.8	123.1	30.3	1.9	12.4
	Standing	108	90.8	15.6	133.3	23.7	12.3	2.7	2,843	669	1.3	13.0	197.0	52.7	−0.9	13.1
	Visual	7	94.0	10.5	137.8	16.6	12.7	2.6	2,792	721	−5.6	14.7	205.3	33.5	1.1	17.3
6-Iron	Intellectual	17	81.5	12.2	112.7	18.1	15.2	3.2	5,501	1,099	2.1	7.5	153.1	32.7	1.0	6.8
	Sitting	10	57.9	7.0	76.1	11.6	17.0	4.5	3,945	1,105	6.0	4.8	76.9	25.6	2.2	6.9
	Standing	108	77.0	12.0	106.4	18.5	15.3	3.1	5,207	1,087	0.6	8.0	139.0	35.0	−0.8	7.8
	Visual	7	78.8	8.7	110.5	15.3	15.4	2.5	4,725	674	−2.5	8.2	151.3	29.4	0.8	7.3
Pitching Wedge	Intellectual	17	75.1	11.4	89.5	16.8	25.5	4.6	7,197	2,034	−0.2	4.6	112.0	27.6	−0.5	4.0
	Sitting	10	53.6	6.3	57.8	8.8	28.7	4.6	3,778	1,051	7.5	11.1	55.3	18.6	0.3	4.0
	Standing	108	71.4	11.0	84.9	15.3	25.3	4.6	6,600	1,729	0.0	5.9	102.0	26.5	−0.1	4.5
	Visual	7	72.2	7.8	88.8	13.2	26.5	4.5	5,225	1,492	0.6	6.3	114.0	27.4	4.1	4.8
Driver	Intellectual	17	0.7	0.3	2.1	0.9	1.4	0.9	402	247	6.4	3.4	7.4	4.7	11.5	4.6
	Sitting	10	0.6	0.3	1.5	0.7	1.7	0.6	395	170	6.3	2.4	6.4	2.9	5.6	3.8
	Standing	108	0.6	0.3	1.7	1.0	1.4	0.7	365	216	6.0	3.8	6.3	4.0	10.1	6.2
	Visual	7	0.9	0.6	2.5	0.7	1.7	0.5	317	91	8.8	5.4	12.3	7.4	10.6	4.9
6-Iron	Intellectual	17	0.7	0.3	2.2	1.0	1.0	0.6	476	296	4.4	3.1	5.0	2.8	6.2	1.7
	Sitting	10	0.7	0.6	3.2	2.1	2.7	0.9	585	200	5.5	2.3	11.3	7.5	3.1	2.2
	Standing	108	0.5	0.3	2.6	1.8	1.1	0.7	475	283	4.3	3.2	6.0	4.9	6.4	3.3
	Visual	7	0.6	0.4	2.6	1.5	1.0	0.4	408	293	5.0	3.3	6.9	3.1	5.4	1.8
Pitching Wedge	Intellectual	17	0.8	0.3	2.0	1.2	2.4	1.5	807	518	3.1	2.2	3.6	1.1	3.1	1.4
	Sitting	10	0.8	0.6	3.7	2.4	4.2	1.7	856	548	7.8	6.6	6.4	3.9	2.4	1.1
	Standing	108	0.7	0.4	2.1	1.2	2.1	1.7	708	383	3.9	3.7	4.1	3.8	3.4	1.7
	Visual	7	0.5	0.2	3.1	1.2	3.9	2.9	736	400	4.9	2.1	4.5	1.2	5.8	1.6

The intra-individual median absolute deviation values for the outcome measures for the playing style categories are provided in Table 2. The GLM for carry distance variability was statistically significant (*F* = 8.5, *p* < 0.001, *f^2^* = 0.10). The Visual playing style [*B* = 2.4, SE = 1.0, *p* = 0.013, 95% CI (0.5 4.3), partial *f*^2^ = 0.01] and the Sitting playing style were associated with higher carry distance variability [*B* = 2.6, SE = 0.8, *p* = 0.002, 95% CI (1.0 4.2), partial *f*^2^ = 0.02]. The GLM for offline distance variability was statistically significant (*F* = 43.3, *p* < 0.001, *f^2^* = 0.52). The Sitting playing style was associated with a lower variability in Offline Distance [*B* = −2.9, SE = 0.7, *p* < 0.001, 95% CI (−4.4 −1.5), partial *f*^2^ = 0.04].

The intra-individual median values for the outcome measures for the different Sport Classes are provided in Table 3. Although the GLM for carry distance performance was statistically significant (*F* = 79.2, *p* < 0.001, *f^2^* = 0.99), the coefficients for the Standing Sport Classes were not statistically significant (the coefficients for club in this model were statistically significant). The GLM examining offline distance performance was not statistically significant (*F* = 0.9, *p* = 0.438, *f^2^* = 0.01).

**Table 3:** Performance (intra-individual median) measures for sport class for the three clubs tested.

Club	Sport Class	N	Clubhead Speed (mph)		Ball Speed (mph)		Ball Launch Angle (degrees)		Ball Spin Rate (rpm)		Ball Spin Axis (degrees)		Carry Distance (yards)		Offline Distance (yards)	
			Mean	SD	Mean	SD	Mean	SD	Mean	SD	Mean	SD	Mean	SD	Mean	SD
Driver	Intellectual 1	12	97.0	12.7	142.2	19.0	10.6	2.8	2,942	687	−1.3	11.1	214.8	38.8	−4.3	13.4
	Intellectual 2	5	92.6	23.8	131.3	37.4	12.9	4.1	3,207	544	11.4	9.5	185.7	70.0	5.1	6.4
	Sitting 1	5	62.7	6.0	92.4	9.9	13.7	3.0	2,430	552	6.3	20.4	106.7	23.8	−2.2	13.0
	Sitting 2	5	72.0	8.4	105.2	12.4	14.1	1.8	3,025	613	6.9	3.2	139.4	28.8	5.9	11.7
	Standing 1	14	89.4	14.6	130.9	23.3	12.2	2.2	2,652	471	−2.7	12.3	189.9	53.9	2.6	7.8
	Standing 2	47	88.7	15.8	130.5	24.2	12.7	2.6	2,797	678	3.5	13.8	192.8	56.0	−0.5	11.3
	Standing 3	47	93.4	15.6	136.8	23.4	11.9	2.9	2,945	704	0.3	12.1	203.3	49.5	−2.2	15.8
	Visual 1	1	85.0	0.0	123.4	0.0	13.2	0.0	2,638	0	−21.3	0.0	163.6	0.0	−9.1	0.0
	Visual 2	6	95.5	10.7	140.2	16.8	12.7	2.8	2,818	787	−3.0	14.2	212.3	30.6	2.8	18.3
6-Iron	Intellectual 1	12	82.5	9.6	115.3	13.9	14.5	3.0	5,631	1,036	2.0	5.9	159.4	25.3	0.2	7.4
	Intellectual 2	5	79.3	18.3	106.3	26.7	16.7	3.3	5,188	1,307	2.4	11.4	137.9	46.0	2.9	5.2
	Sitting 1	5	53.9	5.3	69.6	9.3	17.0	6.3	3,398	1,270	5.8	4.5	62.0	23.9	−0.8	6.1
	Sitting 2	5	61.8	6.5	82.6	10.6	17.0	2.3	4,492	620	6.2	5.7	91.7	19.0	5.2	6.9
	Standing 1	14	76.1	11.0	105.3	18.5	15.8	2.3	5,338	943	−0.1	4.5	139.1	31.9	0.2	6.6
	Standing 2	47	75.4	12.3	104.9	18.8	15.2	2.9	5,070	1,193	1.8	7.2	136.3	37.7	−0.1	7.1
	Standing 3	47	78.8	12.0	108.3	18.3	15.3	3.5	5,304	1,020	−0.5	9.4	141.6	33.7	−1.8	8.7
	Visual 1	1	74.9	0.0	97.8	0.0	18.0	0.0	4,258	0	−3.7	0.0	132.1	0.0	0.0	0.0
	Visual 2	6	79.5	9.3	112.6	15.6	14.9	2.5	4,803	703	−2.3	9.0	154.5	30.8	1.0	8.0
Pitching Wedge	Intellectual 1	12	75.9	9.3	91.2	14.6	25.4	4.8	7,145	2,036	−0.9	3.8	116.1	25.1	−1.2	3.9
	Intellectual 2	5	73.2	16.6	85.5	22.8	25.8	4.7	7,323	2,262	1.6	6.3	102.0	33.6	1.2	4.0
	Sitting 1	5	50.2	5.4	58.5	7.4	26.2	4.1	3,956	1,227	5.1	6.3	54.6	16.5	−1.3	3.9
	Sitting 2	5	56.9	5.6	57.2	10.9	31.3	3.7	3,599	947	9.8	15.0	56.0	22.4	2.0	3.7
	Standing 1	14	70.8	10.3	84.7	15.7	26.3	3.3	6,453	1,587	1.7	4.7	100.7	24.4	1.3	3.3
	Standing 2	47	70.2	11.5	83.9	15.5	25.3	4.3	6,617	1,752	0.4	5.3	101.6	27.3	−0.2	4.2
	Standing 3	47	72.8	10.7	86.0	15.3	25.1	5.2	6,627	1,778	−0.8	6.8	102.9	26.8	−0.3	5.0
	Visual 1	1	66.8	0.0	82.6	0.0	30.4	0.0	3,733	0	2.7	0.0	101.0	0.0	7.5	0.0
	Visual 2	6	73.1	8.2	89.9	14.2	25.9	4.6	5,474	1,467	0.3	6.9	116.2	29.4	3.6	4.9

The intra-individual median absolute deviation values for the outcome measures for the different Sport Classes are provided in Table 4. The GLM for carry distance variability and offline distance variability were statistically significant (*F* = 8.5, *p* < 0.001, *f^2^* = 0.06, and *F* = 43.3, *p* < 0.001, *f^2^* = 0.01 respectively) however, the coefficients for the Standing Sport Classes were not statistically significant.

**Table 4:** Variability (intra-individual median absolute deviation) measures for sport class for the three clubs tested.

Club	Sport Class	N	Clubhead Speed (mph)		Ball Speed (mph)		Ball Launch Angle (degrees)		Ball Spin Rate (rpm)		Ball Spin Axis (degrees)		Carry Distance (yards)		Offline Distance (yards)	
			Mean	SD	Mean	SD	Mean	SD	Mean	SD	Mean	SD	Mean	SD	Mean	SD
Driver	Intellectual 1	12	0.8	0.2	1.9	0.8	1.2	0.5	375	199	6.6	3.5	7.2	4.6	11.0	3.8
	Intellectual 2	5	0.6	0.3	2.4	1.2	2.0	1.5	466	358	5.8	3.4	7.7	5.6	12.9	6.5
	Sitting 1	5	0.4	0.2	1.1	0.5	1.4	0.7	278	63	4.9	2.3	6.9	3.8	2.7	1.1
	Sitting 2	5	0.7	0.4	1.9	0.7	2.1	0.4	512	163	7.7	1.8	5.8	2.1	8.5	3.1
	Standing 1	14	0.5	0.2	1.6	0.7	1.5	0.6	337	200	6.6	5.1	6.2	4.7	10.7	7.5
	Standing 2	47	0.6	0.2	1.6	0.9	1.5	0.8	303	181	5.6	3.0	5.7	3.7	9.4	5.1
	Standing 3	47	0.7	0.4	1.8	1.1	1.3	0.7	436	235	6.3	4.0	6.9	4.1	10.6	6.7
	Visual 1	1	0.9	0.0	2.6	0.0	2.0	0.0	373	0	15.4	0.0	19.1	0.0	8.6	0.0
	Visual 2	6	0.9	0.7	2.5	0.8	1.6	0.6	307	96	7.7	5.0	11.2	7.5	10.9	5.2
6-Iron	Intellectual 1	12	0.7	0.2	2.0	0.8	0.9	0.6	483	303	4.4	3.2	3.9	1.7	6.3	1.5
	Intellectual 2	5	0.6	0.3	2.7	1.4	1.1	0.8	458	312	4.3	3.0	7.6	3.3	6.1	2.5
	Sitting 1	5	0.8	0.8	4.2	2.4	2.7	1.1	508	153	7.2	1.6	14.0	7.8	2.4	1.4
	Sitting 2	5	0.6	0.4	2.2	1.3	2.6	0.8	662	228	3.7	1.5	8.7	7.0	3.8	2.7
	Standing 1	14	0.5	0.3	2.0	0.9	1.1	0.5	457	276	3.6	1.7	4.7	2.2	6.1	3.3
	Standing 2	47	0.5	0.2	2.7	2.1	1.1	0.7	488	301	4.6	4.2	6.4	6.0	6.0	3.3
	Standing 3	47	0.6	0.3	2.7	1.7	1.2	0.7	468	272	4.3	2.4	6.0	4.2	6.8	3.4
	Visual 1	1	0.6	0.0	4.8	0.0	0.9	0.0	1,021	0	7.5	0.0	11.0	0.0	6.8	0.0
	Visual 2	6	0.6	0.4	2.2	1.2	1.0	0.5	306	124	4.6	3.4	6.2	2.8	5.2	1.8
Pitching Wedge	Intellectual 1	12	0.8	0.3	1.9	1.2	2.4	1.5	916	549	3.3	2.6	3.7	1.1	3.0	1.5
	Intellectual 2	5	0.6	0.2	2.4	1.1	2.2	1.6	544	348	2.6	0.7	3.4	1.2	3.4	1.3
	Sitting 1	5	0.9	0.8	2.8	1.4	4.4	2.3	778	232	5.7	3.9	5.6	2.4	1.8	1.0
	Sitting 2	5	0.8	0.6	4.6	2.9	3.9	1.1	934	779	9.9	8.5	7.3	5.2	3.0	0.8
	Standing 1	14	0.7	0.3	1.8	1.4	1.7	1.1	589	173	3.7	3.7	3.6	2.6	3.1	1.6
	Standing 2	47	0.5	0.3	2.0	0.9	2.0	1.3	716	408	3.6	2.5	3.8	2.5	3.3	1.7
	Standing 3	47	0.8	0.5	2.3	1.4	2.3	2.1	734	402	4.2	4.6	4.6	5.1	3.6	1.8
	Visual 1	1	0.7	0.0	4.2	0.0	6.8	0.0	1,251	0	8.0	0.0	5.7	0.0	5.5	0.0
	Visual 2	6	0.5	0.2	2.9	1.2	3.4	2.8	650	361	4.4	1.8	4.3	1.1	5.9	1.7

The intra-individual median values for the outcome measures for the amputee golfers are provided in Table 5. The GLM for carry distance performance was statistically significant (*F* = 11.1, *p* < 0.001, *f^2^* = 0.88) however, the coefficients for the amputation location and side were not statistically significant. For offline distance performance, the GLM was not statistically better than a constant model (*F* = 0.6, *p* = 0.711, *f^2^* = 0.05).

**Table 5:** Performance (intra-individual median—top) and variability (intra-individual median absolute deviation—bottom) for amputation location and Side for the three clubs tested.

Club	Amputation Location and Side	N	Clubhead Speed (mph)		Ball Speed (mph)		Ball Launch Angle (degrees)		Ball Spin Rate (rpm)		Ball Spin Axis (degrees)		Carry Distance (yards)		Offline Distance (yards)	
			Mean	SD	Mean	SD	Mean	SD	Mean	SD	Mean	SD	Mean	SD	Mean	SD
Driver	Lead Leg Above Knee	5	95.2	23.2	141.4	34.8	10.2	2.4	2,794	523	10.6	12.4	208.4	79.3	3.1	11.6
	Lead Leg Below Knee	8	97.9	17.3	143.3	25.5	12.2	4.1	3,122	778	−0.7	13.8	219.3	47.2	4.5	9.0
	Trail Leg Above Knee	3	93.2	29.0	136.8	45.0	12.8	2.4	3,057	1,267	6.1	7.5	207.0	98.6	3.4	9.3
	Trail Leg Below Knee	7	95.7	20.7	141.6	30.9	10.8	1.4	3,056	584	5.1	5.9	212.8	65.1	−0.1	10.6
6-Iron	Lead Leg Above Knee	5	81.0	16.5	114.1	24.9	14.2	2.4	5,949	1,031	5.7	9.3	151.4	48.7	−2.2	6.2
	Lead Leg Below Knee	8	83.3	12.8	114.5	18.9	13.1	3.5	5,322	1,150	−1.4	8.0	154.3	38.6	1.0	5.1
	Trail Leg Above Knee	3	78.0	22.0	108.0	34.5	13.8	2.1	5,975	1,294	2.4	1.2	136.1	69.3	2.9	4.8
	Trail Leg Below Knee	7	79.7	15.7	107.5	24.8	16.4	4.4	5,404	410	4.3	10.7	141.6	42.0	2.8	10.3
Pitching Wedge	Lead Leg Above Knee	5	75.5	15.1	87.8	21.5	26.8	5.0	7,202	1,909	4.2	6.8	107.2	37.1	−2.1	3.1
	Lead Leg Below Knee	8	76.0	11.5	89.5	16.1	21.8	5.5	6,504	2,359	−1.3	5.8	110.1	34.0	1.1	2.5
	Trail Leg Above Knee	3	73.1	18.9	86.9	30.5	25.9	2.3	6,366	1,392	0.4	5.7	105.8	52.3	2.6	3.5
	Trail Leg Below Knee	7	74.1	13.0	85.6	20.5	26.8	7.4	5,881	2,019	1.4	6.5	104.0	31.5	0.2	4.8
Driver	Lead Leg Above Knee	5	0.6	0.2	1.4	0.9	1.3	0.7	255	128	5.8	5.0	6.5	3.2	10.7	7.1
	Lead Leg Below Knee	8	0.7	0.5	1.8	0.8	1.8	0.7	569	408	8.5	6.7	9.6	7.1	12.1	3.5
	Trail Leg Above Knee	3	0.5	0.1	1.4	0.0	1.4	0.4	343	67	4.6	0.3	5.0	1.6	8.9	5.7
	Trail Leg Below Knee	7	0.5	0.3	1.3	0.6	0.9	0.5	403	217	4.1	2.3	6.1	2.8	9.8	9.2
6-Iron	Lead Leg Above Knee	5	0.7	0.4	2.0	1.0	0.9	0.7	468	231	2.3	0.7	2.9	1.7	5.3	5.0
	Lead Leg Below Knee	8	0.6	0.5	3.2	2.0	1.4	1.1	573	287	4.9	2.5	9.5	5.9	6.4	2.6
	Trail Leg Above Knee	3	0.6	0.2	2.4	1.8	0.9	0.8	301	149	4.6	3.8	3.7	3.2	4.4	4.4
	Trail Leg Below Knee	7	0.5	0.3	3.0	1.1	1.3	0.6	597	356	4.2	2.4	5.0	2.9	7.0	3.7
Pitching Wedge	Lead Leg Above Knee	5	0.5	0.2	1.6	0.5	1.9	0.4	688	206	4.4	4.4	3.1	1.4	3.3	2.1
	Lead Leg Below Knee	8	1.0	0.6	2.2	1.0	2.7	2.5	796	504	6.4	8.2	6.2	7.6	3.7	0.9
	Trail Leg Above Knee	3	0.7	0.3	2.4	2.1	2.5	2.1	783	375	2.9	2.7	2.8	2.3	2.0	1.0
	Trail Leg Below Knee	7	0.9	0.5	2.7	2.5	2.5	1.9	718	306	5.3	6.3	4.0	2.7	3.2	2.4

The intra-individual median absolute deviation values for the outcome measures for the amputee golfers are provided in Table 5. The GLM for carry distance variability was not statistically significant (*F* = 0.6, *p* = 0.008, *f^2^* = 0.27). The GLM for offline distance variability was statistically significant (*F* = 7.2, *p* < 0.001, *f^2^* = 0.57) however, the coefficients for the amputation location and side were not statistically significant.

Full details for the GLM's are provided in Supplementary Material S2. Percentile data, including median values, for outcome variables are provided in Supplementary Material S3.

## Discussion

This study provides normative performance data for a significant proportion of the worldwide population of golfers with disabilities. A 2023 study reported that there were 1734 golfers with disabilities registered with EDGA, and 931 of these were within Northern Europe, North America, or Oceania (the locations for the study). Therefore, the sample in this study represents around 15.3% of the population of interest.

The proportion of female golfers (19.0%) was higher in this data set than in the general population of golfers with disabilities (8.4%) (14). This is important as sports research can overrepresent male participants (15). 76.1% of the participants in this data set were in one of the Standing Sport Classes (Standing 1, Standing 2, or Standing 3). In comparison, 79.4% of golfers in a study of the EDGA database had a reported disability of either amputation (above knee, below knee, or other), orthopedic, or neurological (14) – the majority of whom would play in a Standing Sport Class. Intellectual and Visual impairments represented 12.0% and 4.9% of this data set, whereas they accounted for 4.8% and 5.5% of the general population, respectively. Seated golfers represented 7.0% of this data set, while golfers with reported Spinal Cord Lesion (of which the majority would play in a Seated Class) represented 5.5% of the general population.

Tweedy, Mann and Vanlandewijck (16) detailed the key stages of research required for evidence-based classification in Paralympic sport. The present study, primarily descriptive in nature, is intended to support Stage 2; the development of a theoretical model to identify the determinants of performance in the sport (17). Whilst golf performance is multifaceted, the measures studied in this investigation (clubhead presentation, ball launch, and shot outcome measures), and the variability of these measures, have previously been linked to skill level in able-bodied golfers (18). However, they have not been previously reported in golfers with disabilities. The aim of this study was to determine normative data for competitive golfers with disabilities and examine the relationships between Sport Classes and swing performance. It is hoped that the descriptive data contained within will prove valuable to future researchers undertaking rigorous, process-focused research on the individual Sport Classes examined here.

The largest finding from this research was that the sitting playing style displayed the largest differences in performance amongst the groups studied. These players had lower carry distance (60.9 yards lower median distance across all clubs, partial *f^2^* = 0.17; medium effect), higher carry distance variability (2.6 yards greater intra-individual median absolute deviation, partial *f^2^* = 0.02; small effect), but also lower variability in offline distance variability (4.3 yards lower intra-individual median absolute deviation, partial *f^2^* = 0.04; small effect). The lower variability in offline distance, which would normally reflect better performance, is likely because these players do not hit the ball as far as the other golfers studied. The same angle from the target line will result in less offline distance for a shorter shot and thus, it does not reflect a higher performance as it might otherwise.

Clubhead speed and total distance are established key indicators of performance (11). That is, the closer a golfer is to the hole when playing their second shot, the more likely it is that they will have a lower score (19). The current study suggests that golfers in the seated class are at a relative disadvantage as they do not hit the ball as far as other golfers with disabilities.

These findings are not unsurprising given the important of segmental sequencing in developing high clubhead speeds in golf (20). Seated golfers have impaired ranges of motion, likely due both to the constraint of being seated and the impairment itself, and this creates a fundamental limitation in the development of speed in the golf swing. The research by Hanks et al. (21) demonstrated this limitation in wheelchair lacrosse players, who exhibited lower rotational velocities and ranges of motion during overhead throwing. When playing on the course, this disadvantage may be further compounded by course set up, natural variations in the course and position of bunkers. These findings support the implementation of adaptive measures such as alternate tee markers for seated golfers to ensure these golfers are not unfairly penalised in competition, and that golf ability and training is the main driver of enhanced performance.

The visual playing style had higher carry distance variability (2.4 yards greater intra-individual median absolute deviation respectively, partial *f^2^* = 0.01; small effect). There were no statistical differences in the visual golfers carry distance performance, which suggests these golfers' primary impairment is one of distance control. This is a potentially novel finding which deserves further investigation and may point to a need to consider different adaptive measures for these golfers in competition. Shorter hole lengths via modifications to tee markers are likely to be an insufficient adaptation for these golfers and may not allow these golfers to effectively compete with other golfers with disabilities due to the nature of the visual impairment. It has been suggested that existing methods of visual impairment categorisation do not fully achieve their purpose (22) and further research is required to better understand the effect visual impairment has on performance in golf.

The wide range of performance differences among the cohort were particularly striking. For instance, the maximum median carry distance with a driver club was 292.0 yards (an athlete in the Standing 3 Sport Class), whereas the minimum within the same Sport Class was just 93.9 yards (a range of 198.1 yards). The intention of Sport Classes is to differentiate based on impairment level, not trained ability. This is so that athletes who enhance their ability through effective training are not moved to a new class to compete with athletes who have less impairment in that activity (2). It is possible that the wide range of performance within some Sport Classes reflects a wide range of skill of the golfers sampled at these events, but it may also reflect a range of impairment levels within categories. The results of this study cannot differentiate between skill and impairment related differences in performance. This does however identify a key area for future research which should consider intra-class impairment. Specifically, it would be valuable to consider the objective degree of impairment (for instance, via measurements of strength or range of motion) within these Sport Classes.

### Limitations

This study may be classified as “product-focused” research (23) and, as such, is limited in the ability to support evidence-based classification in disability sport (2). Product-focused research evaluates the relationships between and within formal categories resulting from classification but is limited because it is not possible to determine whether differences are determined by the athletes impairment or sporting ability. However, this evaluation of performance across categories currently used in elite golf for golfers with disabilities does provide a useful base for future research.

There was also a limited sample size in several of the Sport Classes due to the extremely limited population of golfers within these categories. Whilst this limits the generalizability of these specific findings, the data should provide valuable insight into the performance of these golfers which would otherwise be unavailable. The statistical power of the results may be negatively impacted by the number of golfers recruited in each Sport Class; however, the population of interest is very small and data on these golfers exceedingly sparce. As such, the data still provide a valuable resource for developing hypotheses within the research area.

Apart from the finding that seated golfers have significant limitations in carry distance performance, the other observations in this study had small effect sizes. The practical significance of these effects is unclear but encourage future process-focused research in these areas. One such area for future research is the lack of difference in performance between golfers in the three standing Sport Classes.

This research evaluated performance in ball-striking ability with different clubs. Whilst this is a key feature of success in golf, overall performance is influenced by several other factors, including putting, shot selection, and course management skills. It is possible that the nature of impairment for some of the golfers in the study would not be observed in a ball-striking task but impairs their scoring ability in other ways. This should be considered in future research.

### Policy implications

The methods and findings in this study provide a basis for future research aimed at evidence-based classification in disability golf. Consideration could be given to the mechanisms behind the impairments observed in this study or to intra-class differences in impairment. The findings also provide some justification for event organizers to explore the possibility of alternative tee markers for seated golfers and alternative adaptations for visually impaired golfers.

## Conclusion

This study is the first known objective analysis of ball-striking abilities across different disability classes in competitive golfers with disabilities. The findings confirm the known views on the wide range of ability in key performance variables, whilst providing normative data by disability class for use by the wider golf industry and shedding some light on key differences in ability by class. The main observed difference between the groups was between the sitting golfers and the other playing categories: Sitting golfers displayed significantly lower carry distance across all clubs. The normative data provided herewith will provide valuable comparative data and could form the basis for hypothesis generation for future research.

## Data Availability

The raw data supporting the conclusions of this article will be made available by the authors, without undue reservation.
