# Supplementary material for: Performance measures across playing categories in competitive golfers with disabilities: a cross-sectional study
Source: Front Sports Act Living. 2026 May 7;8:1748973. doi: 10.3389/fspor.2026.1748973 (PMC13189902; doi:10.3389/fspor.2026.1748973)
Supplement: Supplementary file 1 [file Datasheet1.docx]

# Supplement 1

## Sport Class Explained

The following explanation of the EDGA Sport Classes was written by Dr Roger Hawkes (Director of Eligibility, EDGA), Dr Will Wynter Bee (Chief Medical Officer, EDGA) and Mark Smith (Training Lead, EDGA). It has been included here with permission.

### Intellectual 1:

Players with intellectual impairment and/or Downs Syndrome.

(Virtus ii1 and ii2).

Players have to work hard to understand and apply training techniques and competition strategies, especially on busy competition courses.

### Intellectual 2:

Players with Autistic Spectrum Disorder (ASD).

(Virtus ii3)

Players have to work hard to choose an appropriate shot in a competition environment, especially in changing conditions such as weather and large spectator crowds.

### Sitting 1:

Players with impaired trunk control, lack of consistent static sitting balance and no independent standing balance. Play seated for the entire round.

e.g. High SCL (T12 and above), complete paralysis (e.g. MS/stroke)

Players have difficulty with core control and so play seated and are likely to hold on with one hand and play with the other.

### Sitting 2:

Players with active trunk control, dynamic sitting balance, minimal unsupported standing balance and unable to play standing without support. Play seated for the entire round.

e.g. Low complete SCL (L1 and below), ataxia involving more than one limb, bilateral lower limb amputees, moderate paralysis (e.g., MS/stroke)

Players have core control, but still play seated, and can stabilise and generate reasonable power and are likely to grip the club with two hands.

### Standing 1:

Players with impaired static standing balance with multiple limb impairments or severe single lower limb impairments, such as standing on one leg with no prosthesis. Likely to lose balance during or after the shot-making sequence. Players with significant bilateral arm impairments requiring the use of long-shafted clubs. Very likely buggy allocation.

e.g. Incomplete High SCL, single above knee amputees {no prosthesis), severe bilateral lower limb impairments and/or multiple limb amputations (e.g., arm and leg, two legs {at least one being above knee} or three limbs), severe hemiplegia or bilateral phocomelia, severe musculoskeletal weakness, spasticity, or ataxia.

Players with impaired standing balance with more than one limb affected (amputations, weakness or altered muscle tone). Will usually have a buggy allocation

### Standing 2:

Players with impaired dynamic standing balance, but able to stand on two legs, with prosthetic limbs if required. Able to maintain standing balance throughout and after the shot-making sequence. Likely buggy allocation.

e.g. Incomplete Low SCL, more extensive single limb amputations (e.g., above knee {with prosthesis}, above and below elbow), bilateral below knee amputees, mild bilateral leg weakness, stiffness or spasticity, less severe hemiplegia (playing two-handed) extensive reduced spinal ROM involving neck AND back rotation.

Players can stand unaided, but still have significant balance impairments related to playing golf. Likely to be more limited to a single limb (arm or leg) or mild multi-limb (both legs) or spine.

### Standing 3:

Players with good dynamic standing balance and mobility, able to grip the club with two hands and swing without losing balance or grip. Usually walk the course using a golf cart or carrying clubs. Less likely buggy indication

e.g. Single below knee amputees, short stature, mild ataxia affecting one limb only, mild grip issues (play two handed), reduced spinal ROM involving back OR neck rotation.

Players with almost full function and able to correct for minor instability in balance, body rotation, range of movement or size. These players normally walk the course and can carry their cubs or use a cart, but not normally a buggy.

### Visual 1:

Totally blind. (B1)

Visual 1 is for players with a near-total visual impairment. All play with a fully sighted guide to assist with shot direction and distance.

### Visual 2:

Significant visual impairment. (B2, B3)

Players with a significant visual impairment. Most able to make out the ball when it is at their feet.
