# Supplementary material for: Performance measures across playing categories in competitive golfers with disabilities: a cross-sectional study
Source: Front Sports Act Living. 2026 May 7;8:1748973. doi: 10.3389/fspor.2026.1748973 (PMC13189902; doi:10.3389/fspor.2026.1748973)
Supplement: Supplementary file 2 [file Datasheet2.docx]

# Supplement 2

## Generalized Linear Models

### Carry Distance Performance (intra-individual median of Carry Distance) for playing style

F-statistic vs. constant model: 102.0, **p-value < 0.001**, *f^2^* = 1.21

|  |  | Estimate | 95% CI | Standard Error | t Statistic | p Value | Partial *f*^2^ |
| --- | --- | --- | --- | --- | --- | --- | --- |
|  | (Intercept) | 195.7 | [189.1 203.4] | 3.4 | 58.23 | <0.001 | 8.08 |
| Sport Class | Visual | 10.9 | [-6.0 27.7] | 8.6 | 1.27 | 0.206 | <0.01 |
|  | Sitting | -60.9 | [-75.2 -46.6] | 7.3 | -8.38 | <0.001 | 0.17 |
|  | Intellectual | 11.1 | [-0.2 22.4] | 5.7 | 1.93 | 0.054 | 0.01 |
| Club | Iron | -56.4 | [-65.3 -47.5] | 4.5 | -12.47 | <0.001 | 0.37 |
|  | Wedge | 92.8 | [-101.7 -83.9] | 4.5 | -20.52 | <0.001 | 1.00 |

For example, to calculate the estimated carry distance performance for a golfer in the sitting playing style, with the 6-iron club, the model would be: Carry distance = 195.7 - 60.9 – 56.4 = 78.4 yards (actual average performance = 76.9 yards). The standing category and driver club are the reference categories and as such the estimated carry distance performance for this combination is 195.7 (actual average performance = 197.0 yards).

### Carry Distance Variability (intra-individual median absolute deviation of Carry Distance) for playing style

F-statistic vs. constant model: 8.5, **p-value < 0.001**, *f^2^* = 0.10

|  |  | Estimate | 95% CI | Standard Error | t Statistic | p Value | Partial *f*^2^ |
| --- | --- | --- | --- | --- | --- | --- | --- |
|  | (Intercept) | 6.4 | [5.7 7.2] | 0.4 | 16.98 | **<0.001** | 0.69 |
| Sport Class | Visual | 2.4 | [0.5 4.3] | 1.0 | 2.50 | 0.013 | 0.01 |
|  | Sitting | 2.6 | [1.0 4.2] | 0.8 | 3.13 | 0.002 | 0.02 |
|  | Intellectual | -0.1 | [-1.4 1.1] | 0.6 | -0.21 | 0.834 | <0.01 |
| Club | Iron | -0.4 | [-1.4 0.6] | 0.5 | -0.81 | 0.419 | <0.01 |
|  | Wedge | -2.5 | [-3.5 -1.5] | 0.5 | -4.86 | <0.001 | 0.06 |

### Offline Distance Performance (intra-individual median of Offline Distance) for playing style

F-statistic vs. constant model: 0.7, p-value = 0.651, *f^2^* = 0.01

|  |  | Estimate | 95% CI | Standard Error | t Statistic | p Value | Partial *f*^2^ |
| --- | --- | --- | --- | --- | --- | --- | --- |
|  | (Intercept) | -1.0 | [-2.5 0.6] | 0.8 | -1.19 | 0.235 | <0.01 |
| Sport Class | Visual | 2.6 | [-1.4 6.6] | 2.0 | 1.27 | 0.205 | <0.01 |
|  | Sitting | 2.0 | [-1.4 5.5] | 1.7 | 1.17 | 0.242 | <0.01 |
|  | Intellectual | 0.2 | [-2.5 2.9] | 1.4 | 0.15 | 0.884 | <0.01 |
| Club | Iron | 0.4 | [-1.8 2.5] | 1.1 | 0.33 | 0.739 | <0.01 |
|  | Wedge | 0.8 | [-1.3 2.9] | 1.1 | 0.73 | 0.469 | <0.01 |

### Offline Distance Variability (intra-individual median absolute deviation of Offline Distance) for playing style

F-statistic vs. constant model: 43.3, **p-value < 0.001**, *f^2^* = 0.52

|  |  | Estimate | 95% CI | Standard Error | t Statistic | p Value | Partial *f*^2^ |
| --- | --- | --- | --- | --- | --- | --- | --- |
|  | (Intercept) | 10.1 | [9.4 10.8] | 0.3 | 29.19 | <0.001 | 2.03 |
| Sport Class | Visual | 0.7 | [-1.1 2.4] | 0.9 | 0.75 | 0.455 | <0.01 |
|  | Sitting | -2.9 | [-4.4 -1.5] | 0.7 | -3.91 | <0.001 | 0.04 |
|  | Intellectual | 0.3 | [-0.8 1.5] | 0.6 | 0.55 | 0.579 | <0.01 |
| Club | Iron | -3.9 | [-4.8 -3.0] | 0.5 | -8.36 | <0.001 | 0.17 |
|  | Wedge | -6.5 | [-7.4 -5.6] | 0.5 | -14.04 | <0.001 | 0.47 |

### Carry Distance Performance (intra-individual median of Carry Distance) for Standing Sport Classes

F-statistic vs. constant model: 79.2, **p-value < 0.001**, *f^2^* = 0.99

|  |  | Estimate | 95% CI | Standard Error | t Statistic | p Value | Partial *f*^2^ |
| --- | --- | --- | --- | --- | --- | --- | --- |
|  | (Intercept) | 194.6 | [185.6 203.6] | 4.6 | 42.62 | <0.001 | 5.69 |
| Sport Class | Standing 3 | 5.7 | [-3.6 15.0] | 4.7 | 1.20 | 0.230 | <0.01 |
|  | Standing 1 | -0.3 | [-14.0 13.4] | 7.0 | -0.05 | 0.961 | <0.01 |
| Club | Iron | -58.0 | [-68.6 -47.4] | 5.4 | -10.76 | <0.001 | 0.360 |
|  | Wedge | -95.0 | [-105.6 -84.4] | 5.4 | -17.61 | <0.001 | 0.97 |

### Carry Distance Variability (intra-individual median absolute deviation of Carry Distance) for Standing Sport Classes

F-statistic vs. constant model: 4.7, **p-value = 0.001**, *f^2^* = 0.06

|  |  | Estimate | 95% CI | Standard Error | t Statistic | p Value | Partial *f*^2^ |
| --- | --- | --- | --- | --- | --- | --- | --- |
|  | (Intercept) | 6.1 | [5.1 7.1] | 0.5 | 12.46 | <0.001 | 0.49 |
| Sport Class | Standing 3 | 0.5 | [-0.5 1.5] | 0.5 | 1.05 | 0.296 | <0.01 |
|  | Standing 1 | -0.4 | [-1.9 1.0] | 0.7 | -0.57 | 0.570 | <0.01 |
| Club | Iron | -0.3 | [-1.4 0.9] | 0.6 | -0.46 | 0.643 | <0.01 |
|  | Wedge | -2.2 | [-3.3 -1.0] | 0.6 | -3.74 | <0.001 | 0.04 |

### Offline Distance Performance (intra-individual median of Offline Distance) for Standing Sport Classes

F-statistic vs. constant model: 0.9, p-value = 0.438, *f^2^* = 0.01

|  |  | Estimate | 95% CI | Standard Error | t Statistic | p Value | Partial *f*^2^ |
| --- | --- | --- | --- | --- | --- | --- | --- |
|  | (Intercept) | -0.6 | [-2.7 1.5] | 1.1 | -0.55 | 0.581 | <0.01 |
| Sport Class | Standing 3 | -1.1 | [-3.3 1.0] | 1.1 | -1.05 | 0.295 | <0.01 |
|  | Standing 1 | 1.6 | [-1.5 4.8] | 1.6 | 1.02 | 0.307 | <0.01 |
| Club | Iron | 0.1 | [-2.4 2.5] | 1.2 | 0.05 | 0.959 | <0.01 |
|  | Wedge | 0.8 | [-1.6 3.3] | 1.2 | 0.65 | 0.513 | <0.01 |

### Offline Distance Variability (intra-individual median absolute deviation of Offline Distance) for Standing Sport Classes

F-statistic vs. constant model: 35.3, **p-value < 0.001**, *f^2^* = 0.44

|  |  | Estimate | 95% CI | Standard Error | t Statistic | p Value | Partial *f*^2^ |
| --- | --- | --- | --- | --- | --- | --- | --- |
|  | (Intercept) | 9.7 | [8.8 10.7] | 0.5 | 20.29 | <0.001 | 1.29 |
| Sport Class | Standing 3 | 0.7 | [-0.2 1.7] | 0.5 | 1.47 | 0.143 | <0.01 |
|  | Standing 1 | 0.3 | [-1.1 1.8] | 0.7 | 0.47 | 0.635 | <0.01 |
| Club | Iron | -3.7 | [-4.8 -2.6] | 0.6 | -6.55 | <0.001 | 0.13 |
|  | Wedge | -6.7 | [-7.8 -5.5] | 0.6 | -11.77 | <0.001 | 0.43 |

### Carry Distance Performance (intra-individual median of Carry Distance) for Amputation Location and Side

F-statistic vs. constant model: 11.1, **p-value < 0.001**, *f^2^* = 0.88

|  |  | Estimate | 95% CI | Standard Error | t Statistic | p Value | Partial *f*^2^ |
| --- | --- | --- | --- | --- | --- | --- | --- |
|  | (Intercept) | 213.1 | [182.8 243.4] | 15.2 | 14.06 | <0.001 | 3.14 |
| Amputation Location and Side | Lead Below Knee | 5.5 | [-26.7 37.8] | 16.1 | 0.34 | 0.732 | <0.01 |
|  | Trail Below Knee | -2.9 | [-36.0 30.2] | 16.6 | -0.17 | 0.862 | <0.01 |
|  | Trail Above Knee | -6.1 | [-47.3 35.2] | 20.7 | -0.29 | 0.770 | <0.01 |
| Club | Iron | -65.9 | [-94.8 -37.0] | 14.5 | -4.56 | <0.001 | 0.33 |
|  | Wedge | -106.3 | [-135.2 -77.4] | 14.5 | -7.36 | <0.001 | 0.86 |

### Carry Distance Variability (intra-individual median absolute deviation of Carry Distance) for Amputation Location and Side

F-statistic vs. constant model: 3.5, p-value = 0.008, *f^2^* = 0.27

|  |  | Estimate | 95% CI | Standard Error | t Statistic | p Value | Partial *f*^2^ |
| --- | --- | --- | --- | --- | --- | --- | --- |
|  | (Intercept) | 5.5 | [2.8 8.3] | 1.4 | 3.98 | <0.001 | 0.25 |
| Amputation Location and Side | Lead Below Knee | 4.3 | [1.4 7.3] | 1.5 | 2.91 | 0.005 | 0.13 |
|  | Trail Below Knee | 0.9 | [-2.1 3.9] | 1.5 | 0.59 | 0.558 | 0.01 |
|  | Trail Above Knee | -0.3 | [-4.1 3.5] | 1.9 | -0.15 | 0.879 | <0.01 |
| Club | Iron | -1.3 | [-4.0 1.3] | 1.3 | -1.00 | 0.323 | 0.02 |
|  | Wedge | -2.8 | [-5.5 -0.2] | 1.3 | -2.13 | 0.037 | 0.07 |

### Offline Distance Performance (intra-individual median of Offline Distance) for Amputation Location and Side

F-statistic vs. constant model: 0.6, p-value = 0.711, *f^2^* = 0.05

|  |  | Estimate | 95% CI | Standard Error | t Statistic | p Value | Partial *f*^2^ |
| --- | --- | --- | --- | --- | --- | --- | --- |
|  | (Intercept) | 0.9 | [-3.6 5.4] | 2.2 | 0.39 | 0.699 | <0.01 |
| Amputation Location and Side | Lead Below Knee | 2.6 | [-2.2 7.4] | 2.4 | 1.09 | 0.279 | 0.02 |
|  | Trail Below Knee | 1.4 | [-3.5 6.3] | 2.5 | 0.56 | 0.579 | <0.01 |
|  | Trail Above Knee | 3.4 | [-2.7 9.5] | 3.1 | 1.10 | 0.274 | 0.02 |
| Club | Iron | -1.5 | [-5.8 2.7] | 2.1 | -0.72 | 0.474 | 0.01 |
|  | Wedge | -2.3 | [-6.6 2.0] | 2.1 | -1.08 | 0.285 | 0.02 |

### Offline Distance Variability (intra-individual median absolute deviation of Offline Distance) for Amputation Location and Side

F-statistic vs. constant model: 7.2, **p-value < 0.001**, *f^2^* = 0.57

|  |  | Estimate | 95% CI | Standard Error | t Statistic | p Value | Partial *f*^2^ |
| --- | --- | --- | --- | --- | --- | --- | --- |
|  | (Intercept) | 10.5 | [7.7 13.2] | 1.3 | 7.75 | <0.001 | 0.95 |
| Amputation Location and Side | Lead Below Knee | 0.9 | [-1.9 3.8] | 1.4 | 0.64 | 0.523 | 0.01 |
|  | Trail Below Knee | 0.2 | [-2.7 3.2] | 1.5 | 0.14 | 0.886 | <0.01 |
|  | Trail Above Knee | -1.4 | [-5.0 2.3] | 1.8 | -0.74 | 0.461 | 0.01 |
| Club | Iron | -4.6 | [-7.2 -2.0] | 1.3 | -3.57 | <0.001 | 0.20 |
|  | Wedge | -7.4 | [-10.0 -4.9] | 1.3 | -5.78 | <0.001 | 0.53 |
