# Supplementary material for: Performance measures across playing categories in competitive golfers with disabilities: a cross-sectional study
Source: Front Sports Act Living. 2026 May 7;8:1748973. doi: 10.3389/fspor.2026.1748973 (PMC13189902; doi:10.3389/fspor.2026.1748973)
Supplement: Supplementary file 3 [file Datasheet3.docx]

# Supplement 3

## Percentile Data

### Clubhead speed percentiles for playing style

|  | Playing Style | N | Clubhead Speed (mph) | | | | |
| --- | --- | --- | --- | --- | --- | --- | --- |
|  |  |  | 10th | 25th | 50th | 75th | 90th |
| Driver | Intellectual | 17 | 69.4 | 86.0 | 98.4 | 107.6 | 112.8 |
|  | Sitting | 10 | 56.2 | 62.6 | 66.8 | 72.5 | 79.4 |
|  | Standing | 108 | 71.1 | 79.5 | 92.0 | 102.2 | 109.9 |
|  | Visual | 7 | 82.9 | 86.0 | 90.1 | 101.9 | 110.0 |
| 6-Iron | Intellectual | 17 | 62.8 | 74.7 | 82.1 | 90.8 | 94.3 |
|  | Sitting | 10 | 47.9 | 54.0 | 58.1 | 63.7 | 66.2 |
|  | Standing | 108 | 59.9 | 69.3 | 77.6 | 86.4 | 91.4 |
|  | Visual | 7 | 71.3 | 73.6 | 74.9 | 83.1 | 93.4 |
| Pitching Wedge | Intellectual | 17 | 57.5 | 68.1 | 73.6 | 84.2 | 88.0 |
|  | Sitting | 10 | 44.9 | 50.4 | 54.2 | 56.1 | 61.9 |
|  | Standing | 108 | 55.3 | 63.5 | 72.3 | 80.0 | 85.1 |
|  | Visual | 7 | 65.2 | 67.2 | 68.8 | 76.2 | 85.4 |

### Ball speed percentiles for playing style

|  | Playing Style | N | Ball Speed (mph) | | | | |
| --- | --- | --- | --- | --- | --- | --- | --- |
|  |  |  | 10th | 25th | 50th | 75th | 90th |
| Driver | Intellectual | 17 | 99.8 | 120.4 | 145.8 | 157.2 | 163.8 |
|  | Sitting | 10 | 82.0 | 91.3 | 98.3 | 107.3 | 115.5 |
|  | Standing | 108 | 104.3 | 115.7 | 134.4 | 151.6 | 162.4 |
|  | Visual | 7 | 121.0 | 124.8 | 130.9 | 150.7 | 162.8 |
| 6-Iron | Intellectual | 17 | 82.1 | 104.1 | 115.4 | 126.4 | 131.1 |
|  | Sitting | 10 | 61.0 | 68.8 | 74.6 | 86.7 | 90.9 |
|  | Standing | 108 | 82.0 | 94.1 | 109.3 | 120.8 | 130.1 |
|  | Visual | 7 | 98.2 | 100.5 | 106.3 | 113.6 | 137.1 |
| Pitching Wedge | Intellectual | 17 | 61.5 | 81.5 | 91.6 | 102.4 | 105.2 |
|  | Sitting | 10 | 48.0 | 49.4 | 57.2 | 67.0 | 70.3 |
|  | Standing | 108 | 62.7 | 75.0 | 87.0 | 94.7 | 104.5 |
|  | Visual | 7 | 75.6 | 81.5 | 82.6 | 97.1 | 110.2 |

### Launch angle percentiles for playing style

|  | Playing Style | N | Launch Angle (deg) | | | | |
| --- | --- | --- | --- | --- | --- | --- | --- |
|  |  |  | 10th | 25th | 50th | 75th | 90th |
| Driver | Intellectual | 17 | 7.6 | 9.1 | 10.1 | 14.3 | 16.6 |
|  | Sitting | 10 | 10.2 | 13.4 | 14.7 | 15.1 | 16.4 |
|  | Standing | 108 | 9.0 | 10.5 | 12.0 | 14.0 | 16.1 |
|  | Visual | 7 | 10.2 | 10.9 | 13.1 | 13.2 | 16.9 |
| 6-Iron | Intellectual | 17 | 11.5 | 12.8 | 14.8 | 17.4 | 19.4 |
|  | Sitting | 10 | 11.1 | 14.4 | 16.0 | 19.8 | 23.4 |
|  | Standing | 108 | 11.7 | 13.5 | 15.3 | 17.0 | 18.8 |
|  | Visual | 7 | 11.5 | 13.7 | 15.9 | 17.5 | 18.2 |
| Pitching Wedge | Intellectual | 17 | 20.5 | 22.4 | 25.3 | 26.9 | 31.6 |
|  | Sitting | 10 | 22.6 | 26.6 | 28.3 | 30.9 | 35.2 |
|  | Standing | 108 | 20.2 | 22.5 | 25.4 | 27.9 | 31.1 |
|  | Visual | 7 | 21.0 | 22.3 | 26.2 | 29.8 | 32.6 |

### Spin rate percentiles for playing style

|  | Playing Style | N | Spin Rate (rpm) | | | | |
| --- | --- | --- | --- | --- | --- | --- | --- |
|  |  |  | 10th | 25th | 50th | 75th | 90th |
| Driver | Intellectual | 17 | 2402 | 2619 | 2799 | 3466 | 3892 |
|  | Sitting | 10 | 1883 | 2277 | 2805 | 3115 | 3599 |
|  | Standing | 108 | 2027 | 2395 | 2773 | 3184 | 3818 |
|  | Visual | 7 | 1791 | 2400 | 2638 | 3427 | 3686 |
| 6-Iron | Intellectual | 17 | 3922 | 4843 | 5838 | 6260 | 6697 |
|  | Sitting | 10 | 2404 | 3304 | 3891 | 4716 | 5390 |
|  | Standing | 108 | 3892 | 4332 | 5198 | 5948 | 6625 |
|  | Visual | 7 | 3666 | 4393 | 4821 | 5128 | 5529 |
| Pitching Wedge | Intellectual | 17 | 4221 | 6203 | 7603 | 9065 | 9508 |
|  | Sitting | 10 | 2718 | 3193 | 3511 | 3991 | 5529 |
|  | Standing | 108 | 4006 | 5066 | 6923 | 7943 | 8612 |
|  | Visual | 7 | 3816 | 4228 | 4639 | 6415 | 7552 |

### Spin axis tilt percentiles for playing style

|  | Playing Style | N | Spin Axis Tilt (deg) | | | | |
| --- | --- | --- | --- | --- | --- | --- | --- |
|  |  |  | 10th | 25th | 50th | 75th | 90th |
| Driver | Intellectual | 17 | -17.2 | -3.7 | 4.2 | 7.3 | 17.8 |
|  | Sitting | 10 | -15.3 | 2.5 | 7.9 | 13.3 | 24.4 |
|  | Standing | 108 | -14.2 | -3.4 | 1.5 | 9.3 | 16.9 |
|  | Visual | 7 | -20.7 | -18.0 | -11.1 | 7.5 | 13.5 |
| 6-Iron | Intellectual | 17 | -7.5 | -4.7 | 3.9 | 5.6 | 11.7 |
|  | Sitting | 10 | -1.1 | 3.8 | 6.3 | 9.4 | 12.0 |
|  | Standing | 108 | -8.2 | -3.8 | 1.2 | 5.2 | 9.9 |
|  | Visual | 7 | -13.4 | -8.0 | -3.7 | 3.3 | 9.0 |
| Pitching Wedge | Intellectual | 17 | -6.1 | -4.9 | 0.9 | 2.6 | 5.6 |
|  | Sitting | 10 | -2.1 | -1.8 | 2.7 | 14.1 | 25.9 |
|  | Standing | 108 | -6.4 | -3.8 | 0.0 | 3.6 | 6.7 |
|  | Visual | 7 | -9.1 | -3.7 | 2.7 | 6.0 | 7.2 |

### Carry distance percentiles for playing style

|  | Playing Style | N | Carry Distance (yards) | | | | |
| --- | --- | --- | --- | --- | --- | --- | --- |
|  |  |  | 10th | 25th | 50th | 75th | 90th |
| Driver | Intellectual | 17 | 131.5 | 168.5 | 225.5 | 235.9 | 265.7 |
|  | Sitting | 10 | 81.1 | 98.8 | 121.7 | 150.9 | 163.2 |
|  | Standing | 108 | 125.4 | 158.2 | 202.8 | 238.2 | 261.4 |
|  | Visual | 7 | 166.2 | 178.8 | 205.7 | 235.2 | 251.0 |
| 6-Iron | Intellectual | 17 | 96.1 | 141.8 | 163.4 | 179.6 | 185.0 |
|  | Sitting | 10 | 36.2 | 61.8 | 79.8 | 94.9 | 107.6 |
|  | Standing | 108 | 91.4 | 114.1 | 147.4 | 164.6 | 181.1 |
|  | Visual | 7 | 130.1 | 133.3 | 140.1 | 157.9 | 203.1 |
| Pitching Wedge | Intellectual | 17 | 64.4 | 97.3 | 117.6 | 133.4 | 138.9 |
|  | Sitting | 10 | 32.0 | 40.1 | 53.0 | 76.9 | 80.3 |
|  | Standing | 108 | 64.0 | 83.2 | 106.0 | 119.3 | 136.4 |
|  | Visual | 7 | 91.3 | 96.6 | 101.0 | 125.6 | 160.9 |

### Offline distance percentiles for playing style

|  | Playing Style | N | Offline distance (yards) | | | | |
| --- | --- | --- | --- | --- | --- | --- | --- |
|  |  |  | 10th | 25th | 50th | 75th | 90th |
| Driver | Intellectual | 17 | -19.9 | -11.2 | 1.9 | 7.1 | 12.2 |
|  | Sitting | 10 | -15.2 | -10.5 | 3.2 | 10.6 | 16.8 |
|  | Standing | 108 | -14.3 | -6.8 | 0.5 | 5.3 | 12.8 |
|  | Visual | 7 | -15.9 | -10.3 | -9.1 | 15.7 | 26.4 |
| 6-Iron | Intellectual | 17 | -8.6 | -3.4 | 0.2 | 5.0 | 10.4 |
|  | Sitting | 10 | -6.1 | -2.0 | 1.6 | 3.6 | 12.2 |
|  | Standing | 108 | -8.8 | -4.4 | -0.4 | 3.3 | 9.1 |
|  | Visual | 7 | -6.4 | -5.4 | 0.0 | 7.8 | 11.0 |
| Pitching Wedge | Intellectual | 17 | -7.6 | -2.0 | 0.2 | 2.2 | 3.0 |
|  | Sitting | 10 | -4.7 | -1.3 | -0.2 | 2.6 | 5.5 |
|  | Standing | 108 | -4.9 | -2.2 | 0.1 | 3.1 | 5.0 |
|  | Visual | 7 | -1.9 | 1.1 | 2.4 | 8.1 | 10.2 |

### Clubhead speed percentiles for Sport Class

|  | Sport Class | N | Clubhead Speed (mph) | | | | |
| --- | --- | --- | --- | --- | --- | --- | --- |
|  |  |  | 10th | 25th | 50th | 75th | 90th |
| Driver | Intellectual 1 | 12 | 80.4 | 90.4 | 98.9 | 107.0 | 110.7 |
|  | Intellectual 2 | 5 | 65.9 | 75.2 | 85.9 | 111.7 | 125.7 |
|  | Sitting 1 | 5 | 52.4 | 60.1 | 64.8 | 66.7 | 67.0 |
|  | Sitting 2 | 5 | 59.9 | 66.5 | 72.5 | 77.9 | 82.2 |
|  | Standing 1 | 14 | 74.1 | 81.4 | 89.3 | 100.2 | 109.7 |
|  | Standing 2 | 47 | 66.6 | 77.2 | 91.0 | 100.3 | 109.9 |
|  | Standing 3 | 47 | 73.8 | 80.7 | 95.0 | 104.7 | 114.8 |
|  | Visual 1 | 1 | - | - | 85.0 | - | - |
|  | Visual 2 | 6 | 83.1 | 88.9 | 93.0 | 103.9 | 110.7 |
| 6-Iron | Intellectual 1 | 12 | 71.2 | 76.1 | 83.5 | 90.3 | 94.1 |
|  | Intellectual 2 | 5 | 60.4 | 64.9 | 73.3 | 95.1 | 104.1 |
|  | Sitting 1 | 5 | 45.1 | 51.8 | 54.0 | 57.7 | 58.8 |
|  | Sitting 2 | 5 | 50.7 | 59.4 | 63.7 | 65.9 | 67.0 |
|  | Standing 1 | 14 | 67.2 | 69.4 | 74.8 | 84.8 | 90.6 |
|  | Standing 2 | 47 | 57.3 | 67.0 | 76.7 | 83.5 | 90.8 |
|  | Standing 3 | 47 | 65.2 | 68.9 | 80.9 | 88.0 | 92.8 |
|  | Visual 1 | 1 | - | - | 74.9 | - | - |
|  | Visual 2 | 6 | 71.0 | 73.6 | 76.2 | 84.6 | 94.5 |
| Pitching Wedge | Intellectual 1 | 12 | 64.6 | 69.7 | 76.0 | 83.2 | 87.2 |
|  | Intellectual 2 | 5 | 55.9 | 60.0 | 68.1 | 87.8 | 95.2 |
|  | Sitting 1 | 5 | 41.2 | 48.1 | 51.1 | 53.6 | 55.4 |
|  | Sitting 2 | 5 | 48.6 | 54.0 | 56.1 | 61.1 | 63.6 |
|  | Standing 1 | 14 | 61.7 | 65.4 | 69.7 | 79.4 | 83.7 |
|  | Standing 2 | 47 | 53.4 | 62.4 | 71.6 | 78.9 | 84.2 |
|  | Standing 3 | 47 | 59.5 | 63.4 | 73.9 | 81.4 | 85.8 |
|  | Visual 1 | 1 | - | - | 66.8 | - | - |
|  | Visual 2 | 6 | 65.2 | 68.5 | 70.1 | 77.8 | 86.3 |

### Ball speed percentiles for Sport Class

|  | Sport Class | N | Ball Speed (mph) | | | | |
| --- | --- | --- | --- | --- | --- | --- | --- |
|  |  |  | 10th | 25th | 50th | 75th | 90th |
| Driver | Intellectual 1 | 12 | 115.6 | 133.2 | 145.9 | 155.9 | 162.8 |
|  | Intellectual 2 | 5 | 95.0 | 106.7 | 110.8 | 163.1 | 184.3 |
|  | Sitting 1 | 5 | 75.8 | 87.5 | 97.0 | 98.1 | 100.7 |
|  | Sitting 2 | 5 | 88.2 | 96.6 | 107.3 | 112.6 | 121.5 |
|  | Standing 1 | 14 | 101.5 | 117.9 | 132.5 | 148.7 | 161.4 |
|  | Standing 2 | 47 | 99.7 | 110.5 | 132.7 | 150.2 | 162.1 |
|  | Standing 3 | 47 | 106.3 | 118.4 | 138.7 | 152.6 | 168.0 |
|  | Visual 1 | 1 | - | - | 123.4 | - | - |
|  | Visual 2 | 6 | 121.3 | 129.2 | 136.3 | 153.7 | 164.0 |
| 6-Iron | Intellectual 1 | 12 | 97.9 | 108.1 | 117.8 | 126.0 | 129.5 |
|  | Intellectual 2 | 5 | 79.5 | 82.3 | 102.2 | 129.6 | 141.0 |
|  | Sitting 1 | 5 | 55.9 | 65.6 | 70.4 | 73.6 | 82.0 |
|  | Sitting 2 | 5 | 66.0 | 75.2 | 86.7 | 90.0 | 92.5 |
|  | Standing 1 | 14 | 83.8 | 95.3 | 105.6 | 119.8 | 131.0 |
|  | Standing 2 | 47 | 78.8 | 92.1 | 105.6 | 118.6 | 129.1 |
|  | Standing 3 | 47 | 84.7 | 94.8 | 111.2 | 122.7 | 130.6 |
|  | Visual 1 | 1 | - | - | 97.8 | - | - |
|  | Visual 2 | 6 | 100.0 | 103.0 | 107.4 | 115.3 | 139.9 |
| Pitching Wedge | Intellectual 1 | 12 | 71.2 | 84.9 | 95.0 | 102.1 | 103.6 |
|  | Intellectual 2 | 5 | 60.6 | 64.2 | 86.0 | 105.2 | 112.9 |
|  | Sitting 1 | 5 | 49.4 | 55.1 | 57.3 | 61.6 | 69.9 |
|  | Sitting 2 | 5 | 46.8 | 48.6 | 52.4 | 67.9 | 70.6 |
|  | Standing 1 | 14 | 70.0 | 77.0 | 82.8 | 99.0 | 107.1 |
|  | Standing 2 | 47 | 59.9 | 73.4 | 88.2 | 93.7 | 103.6 |
|  | Standing 3 | 47 | 64.4 | 75.4 | 87.3 | 97.5 | 106.1 |
|  | Visual 1 | 1 | - | - | 82.6 | - | - |
|  | Visual 2 | 6 | 74.8 | 81.4 | 85.7 | 99.5 | 111.5 |

### Launch angle percentiles for Sport Class

|  | Sport Class | N | Launch Angle (deg) | | | | |
| --- | --- | --- | --- | --- | --- | --- | --- |
|  |  |  | 10th | 25th | 50th | 75th | 90th |
| Driver | Intellectual 1 | 12 | 7.8 | 9.0 | 10.1 | 11.4 | 15.0 |
|  | Intellectual 2 | 5 | 7.5 | 9.3 | 14.5 | 15.8 | 17.3 |
|  | Sitting 1 | 5 | 8.9 | 12.3 | 14.7 | 15.3 | 16.8 |
|  | Sitting 2 | 5 | 11.5 | 12.9 | 14.7 | 15.3 | 16.0 |
|  | Standing 1 | 14 | 8.7 | 11.3 | 12.3 | 13.4 | 14.2 |
|  | Standing 2 | 47 | 9.5 | 11.4 | 12.4 | 14.4 | 16.5 |
|  | Standing 3 | 47 | 8.5 | 10.3 | 11.2 | 13.6 | 16.1 |
|  | Visual 1 | 1 | - | - | 13.2 | - | - |
|  | Visual 2 | 6 | 10.1 | 10.8 | 12.1 | 13.2 | 17.3 |
| 6-Iron | Intellectual 1 | 12 | 11.5 | 12.7 | 14.1 | 15.2 | 18.7 |
|  | Intellectual 2 | 5 | 11.4 | 14.9 | 17.8 | 18.9 | 19.6 |
|  | Sitting 1 | 5 | 9.4 | 11.9 | 15.8 | 22.9 | 24.4 |
|  | Sitting 2 | 5 | 14.4 | 15.3 | 16.2 | 19.2 | 19.8 |
|  | Standing 1 | 14 | 12.3 | 14.5 | 15.9 | 17.6 | 18.4 |
|  | Standing 2 | 47 | 11.0 | 13.5 | 15.3 | 16.9 | 19.6 |
|  | Standing 3 | 47 | 12.2 | 13.5 | 15.1 | 16.9 | 18.2 |
|  | Visual 1 | 1 | - | - | 18.0 | - | - |
|  | Visual 2 | 6 | 11.3 | 13.3 | 15.4 | 16.2 | 18.1 |
| Pitching Wedge | Intellectual 1 | 12 | 20.2 | 23.1 | 24.8 | 26.4 | 30.6 |
|  | Intellectual 2 | 5 | 20.7 | 21.9 | 26.4 | 28.3 | 32.8 |
|  | Sitting 1 | 5 | 20.0 | 23.9 | 26.6 | 28.8 | 30.9 |
|  | Sitting 2 | 5 | 27.5 | 28.2 | 30.0 | 35.2 | 35.4 |
|  | Standing 1 | 14 | 21.9 | 23.8 | 26.0 | 28.8 | 31.2 |
|  | Standing 2 | 47 | 19.7 | 22.3 | 26.0 | 28.0 | 30.0 |
|  | Standing 3 | 47 | 20.2 | 21.9 | 24.6 | 27.5 | 31.7 |
|  | Visual 1 | 1 | - | - | 30.4 | - | - |
|  | Visual 2 | 6 | 21.0 | 21.2 | 26.0 | 28.0 | 32.7 |

### Spin rate percentiles for Sport Class

|  | Sport Class | N | Spin Rate (rpm) | | | | |
| --- | --- | --- | --- | --- | --- | --- | --- |
|  |  |  | 10th | 25th | 50th | 75th | 90th |
| Driver | Intellectual 1 | 12 | 2382 | 2477 | 2710 | 3166 | 3925 |
|  | Intellectual 2 | 5 | 2778 | 2794 | 2887 | 3697 | 3963 |
|  | Sitting 1 | 5 | 1783 | 1932 | 2481 | 2871 | 3115 |
|  | Sitting 2 | 5 | 2277 | 2684 | 2831 | 3438 | 3920 |
|  | Standing 1 | 14 | 1861 | 2491 | 2628 | 3031 | 3231 |
|  | Standing 2 | 47 | 2029 | 2323 | 2776 | 3133 | 3770 |
|  | Standing 3 | 47 | 2078 | 2519 | 2789 | 3232 | 4154 |
|  | Visual 1 | 1 | - | - | 2638 | - | - |
|  | Visual 2 | 6 | 1719 | 2362 | 2823 | 3525 | 3706 |
| 6-Iron | Intellectual 1 | 12 | 4280 | 5215 | 5923 | 6268 | 6498 |
|  | Intellectual 2 | 5 | 3821 | 4201 | 4727 | 6310 | 6978 |
|  | Sitting 1 | 5 | 2066 | 2573 | 3304 | 3929 | 5456 |
|  | Sitting 2 | 5 | 3730 | 3972 | 4638 | 4868 | 5324 |
|  | Standing 1 | 14 | 3970 | 5022 | 5364 | 5888 | 6431 |
|  | Standing 2 | 47 | 3624 | 4196 | 4926 | 5963 | 6731 |
|  | Standing 3 | 47 | 3986 | 4620 | 5228 | 5939 | 6707 |
|  | Visual 1 | 1 | - | - | 4258 | - | - |
|  | Visual 2 | 6 | 3647 | 4798 | 4836 | 5220 | 5568 |
| Pitching Wedge | Intellectual 1 | 12 | 4318 | 5890 | 7631 | 8484 | 9421 |
|  | Intellectual 2 | 5 | 4002 | 6086 | 6996 | 9243 | 9769 |
|  | Sitting 1 | 5 | 3032 | 3317 | 3566 | 4274 | 6109 |
|  | Sitting 2 | 5 | 2405 | 2996 | 3456 | 4231 | 4949 |
|  | Standing 1 | 14 | 4717 | 5256 | 6506 | 7604 | 8010 |
|  | Standing 2 | 47 | 4074 | 5105 | 6928 | 8042 | 8692 |
|  | Standing 3 | 47 | 3979 | 5008 | 7100 | 8106 | 8583 |
|  | Visual 1 | 1 | - | - | 3733 | - | - |
|  | Visual 2 | 6 | 4181 | 4462 | 4811 | 6893 | 7634 |

### Spin axis tilt percentiles for Sport Class

|  | Sport Class | N | Spin Axis Tilt (deg) | | | | |
| --- | --- | --- | --- | --- | --- | --- | --- |
|  |  |  | 10th | 25th | 50th | 75th | 90th |
| Driver | Intellectual 1 | 12 | -20.2 | -7.5 | 2.3 | 5.6 | 10.2 |
|  | Intellectual 2 | 5 | 2.8 | 4.0 | 7.2 | 19.9 | 24.4 |
|  | Sitting 1 | 5 | -19.1 | -13.4 | 13.3 | 24.4 | 24.4 |
|  | Sitting 2 | 5 | 2.5 | 5.1 | 6.1 | 9.9 | 10.4 |
|  | Standing 1 | 14 | -15.8 | -7.3 | 1.1 | 3.2 | 9.8 |
|  | Standing 2 | 47 | -9.9 | -1.7 | 2.5 | 11.7 | 20.3 |
|  | Standing 3 | 47 | -15.7 | -7.4 | 0.9 | 7.2 | 15.8 |
|  | Visual 1 | 1 | - | - | -21.3 | - | - |
|  | Visual 2 | 6 | -18.2 | -16.8 | -2.8 | 8.2 | 14.2 |
| 6-Iron | Intellectual 1 | 12 | -5.7 | -3.7 | 3.3 | 5.7 | 9.2 |
|  | Intellectual 2 | 5 | -8.6 | -7.9 | 4.0 | 8.4 | 19.5 |
|  | Sitting 1 | 5 | -1.0 | 2.7 | 6.8 | 9.5 | 9.8 |
|  | Sitting 2 | 5 | -1.1 | 2.6 | 5.9 | 9.6 | 14.3 |
|  | Standing 1 | 14 | -6.8 | -2.1 | -0.4 | 4.0 | 6.8 |
|  | Standing 2 | 47 | -6.8 | -3.2 | 2.1 | 5.4 | 10.2 |
|  | Standing 3 | 47 | -10.8 | -6.5 | 0.2 | 5.9 | 10.6 |
|  | Visual 1 | 1 | - | - | -3.7 | - | - |
|  | Visual 2 | 6 | -14.0 | -9.2 | -2.3 | 4.4 | 9.6 |
| Pitching Wedge | Intellectual 1 | 12 | -6.6 | -4.1 | 0.2 | 1.6 | 2.9 |
|  | Intellectual 2 | 5 | -5.6 | -5.0 | 4.4 | 6.4 | 8.1 |
|  | Sitting 1 | 5 | -1.8 | 0.4 | 3.6 | 9.9 | 14.1 |
|  | Sitting 2 | 5 | -2.5 | -2.0 | 1.7 | 23.8 | 30.0 |
|  | Standing 1 | 14 | -3.8 | -1.4 | 1.2 | 3.4 | 6.9 |
|  | Standing 2 | 47 | -4.9 | -2.8 | -0.1 | 3.5 | 6.2 |
|  | Standing 3 | 47 | -7.5 | -5.8 | -0.2 | 3.9 | 7.7 |
|  | Visual 1 | 1 | - | - | 2.7 | - | - |
|  | Visual 2 | 6 | -9.6 | -4.7 | 1.1 | 7.1 | 7.3 |

### Carry distance percentiles for Sport Class

|  | Sport Class | N | Carry Distance (yards) | | | | |
| --- | --- | --- | --- | --- | --- | --- | --- |
|  |  |  | 10th | 25th | 50th | 75th | 90th |
| Driver | Intellectual 1 | 12 | 160.0 | 191.9 | 225.5 | 236.1 | 265.4 |
|  | Intellectual 2 | 5 | 116.7 | 133.6 | 155.0 | 247.2 | 281.9 |
|  | Sitting 1 | 5 | 79.6 | 81.8 | 119.0 | 124.3 | 130.1 |
|  | Sitting 2 | 5 | 98.8 | 115.5 | 150.9 | 160.3 | 168.9 |
|  | Standing 1 | 14 | 115.1 | 156.9 | 200.8 | 228.4 | 259.8 |
|  | Standing 2 | 47 | 102.8 | 154.4 | 202.4 | 236.9 | 261.7 |
|  | Standing 3 | 47 | 130.4 | 160.9 | 203.2 | 246.3 | 261.6 |
|  | Visual 1 | 1 | - | - | 163.6 | - | - |
|  | Visual 2 | 6 | 177.5 | 185.6 | 207.3 | 243.9 | 251.9 |
| 6-Iron | Intellectual 1 | 12 | 128.4 | 143.5 | 166.7 | 179.8 | 182.3 |
|  | Intellectual 2 | 5 | 87.2 | 93.1 | 142.2 | 179.6 | 188.1 |
|  | Sitting 1 | 5 | 30.5 | 39.1 | 78.2 | 79.6 | 80.0 |
|  | Sitting 2 | 5 | 61.8 | 80.3 | 94.9 | 107.4 | 108.0 |
|  | Standing 1 | 14 | 107.4 | 122.5 | 140.0 | 162.2 | 178.3 |
|  | Standing 2 | 47 | 89.2 | 109.7 | 142.9 | 163.7 | 180.4 |
|  | Standing 3 | 47 | 95.7 | 120.1 | 154.1 | 167.3 | 181.9 |
|  | Visual 1 | 1 | - | - | 132.1 | - | - |
|  | Visual 2 | 6 | 130.3 | 136.9 | 142.5 | 162.2 | 208.2 |
| Pitching Wedge | Intellectual 1 | 12 | 82.4 | 104.2 | 124.0 | 133.7 | 138.6 |
|  | Intellectual 2 | 5 | 61.9 | 71.1 | 107.1 | 129.7 | 141.2 |
|  | Sitting 1 | 5 | 32.4 | 45.7 | 55.9 | 61.8 | 78.4 |
|  | Sitting 2 | 5 | 31.5 | 38.0 | 49.2 | 78.2 | 82.1 |
|  | Standing 1 | 14 | 77.7 | 86.4 | 99.3 | 122.2 | 134.1 |
|  | Standing 2 | 47 | 62.4 | 82.4 | 107.7 | 118.7 | 136.5 |
|  | Standing 3 | 47 | 66.1 | 84.3 | 108.1 | 119.5 | 139.9 |
|  | Visual 1 | 1 | - | - | 101.0 | - | - |
|  | Visual 2 | 6 | 90.8 | 95.3 | 106.4 | 130.0 | 164.8 |

### Offline distance percentiles for Sport Class

|  | Sport Class | N | Offline Distance (yards) | | | | |
| --- | --- | --- | --- | --- | --- | --- | --- |
|  |  |  | 10th | 25th | 50th | 75th | 90th |
| Driver | Intellectual 1 | 12 | -23.6 | -13.1 | -2.6 | 6.9 | 11.6 |
|  | Intellectual 2 | 5 | -3.0 | 0.3 | 6.7 | 8.5 | 13.8 |
|  | Sitting 1 | 5 | -17.8 | -13.9 | -1.7 | 10.5 | 10.6 |
|  | Sitting 2 | 5 | -10.5 | -2.5 | 6.3 | 15.9 | 18.5 |
|  | Standing 1 | 14 | -5.6 | -2.0 | 2.8 | 4.6 | 12.4 |
|  | Standing 2 | 47 | -14.4 | -6.1 | -0.1 | 6.0 | 12.5 |
|  | Standing 3 | 47 | -22.7 | -9.1 | -1.0 | 4.4 | 14.1 |
|  | Visual 1 | 1 | - | - | -9.1 | - | - |
|  | Visual 2 | 6 | -16.6 | -10.3 | -1.2 | 18.3 | 27.4 |
| 6-Iron | Intellectual 1 | 12 | -10.2 | -3.9 | -0.8 | 4.4 | 11.4 |
|  | Intellectual 2 | 5 | -3.3 | -0.7 | 2.3 | 6.3 | 10.5 |
|  | Sitting 1 | 5 | -8.7 | -4.8 | -2.0 | 4.4 | 6.9 |
|  | Sitting 2 | 5 | 1.5 | 1.5 | 1.8 | 7.1 | 17.4 |
|  | Standing 1 | 14 | -6.5 | -4.3 | -1.2 | 3.2 | 8.9 |
|  | Standing 2 | 47 | -8.1 | -4.1 | -0.3 | 3.4 | 9.6 |
|  | Standing 3 | 47 | -14.8 | -4.5 | -0.4 | 3.3 | 7.1 |
|  | Visual 1 | 1 | - | - | 0.0 | - | - |
|  | Visual 2 | 6 | -6.5 | -5.8 | -1.5 | 10.0 | 11.1 |
| Pitching Wedge | Intellectual 1 | 12 | -8.5 | -2.1 | -0.2 | 1.1 | 3.0 |
|  | Intellectual 2 | 5 | -5.1 | -0.9 | 1.8 | 3.6 | 5.7 |
|  | Sitting 1 | 5 | -7.9 | -2.6 | -0.3 | 0.6 | 2.6 |
|  | Sitting 2 | 5 | -1.5 | -1.4 | 1.8 | 4.5 | 7.5 |
|  | Standing 1 | 14 | -2.4 | -1.8 | 1.5 | 4.1 | 5.6 |
|  | Standing 2 | 47 | -4.9 | -2.4 | -0.4 | 2.4 | 4.9 |
|  | Standing 3 | 47 | -6.3 | -2.2 | 0.3 | 3.5 | 5.0 |
|  | Visual 1 | 1 | - | - | 7.5 | - | - |
|  | Visual 2 | 6 | -2.3 | 0.8 | 2.2 | 8.4 | 10.4 |

### Clubhead speed percentiles for Amputation Location and Side

|  | Amputation Location and Side | N |  | | | | |
| --- | --- | --- | --- | --- | --- | --- | --- |
|  |  |  | 10th | 25th | 50th | 75th | 90th |
| Driver | Lead Above Knee | 5 | 55.2 | 89.4 | 101.2 | 106.0 | 116.1 |
|  | Lead Below Knee | 8 | 72.4 | 84.9 | 99.7 | 112.3 | 117.2 |
|  | Trail Above Knee | 3 | 60.7 | 71.2 | 102.8 | 112.8 | 116.2 |
|  | Trail Below Knee | 7 | 69.2 | 81.3 | 95.9 | 113.8 | 122.7 |
| 6-Iron | Lead Above Knee | 5 | 53.8 | 74.8 | 85.0 | 89.3 | 98.4 |
|  | Lead Below Knee | 8 | 62.9 | 75.6 | 86.5 | 90.9 | 96.3 |
|  | Trail Above Knee | 3 | 53.1 | 61.4 | 86.5 | 92.5 | 94.5 |
|  | Trail Below Knee | 7 | 57.2 | 70.3 | 83.2 | 94.2 | 96.9 |
| Pitching Wedge | Lead Above Knee | 5 | 49.5 | 71.5 | 79.1 | 83.3 | 88.7 |
|  | Lead Below Knee | 8 | 58.1 | 67.0 | 80.3 | 83.6 | 87.4 |
|  | Trail Above Knee | 3 | 51.7 | 58.8 | 80.1 | 85.6 | 87.5 |
|  | Trail Below Knee | 7 | 56.2 | 64.7 | 78.8 | 84.6 | 88.9 |

### Ball speed percentiles for Amputation Location and Side

|  | Amputation Location and Side | N | Ball Speed (mph) | | | | |
| --- | --- | --- | --- | --- | --- | --- | --- |
|  |  |  | 10th | 25th | 50th | 75th | 90th |
| Driver | Lead Above Knee | 5 | 80.6 | 134.0 | 152.0 | 157.6 | 168.9 |
|  | Lead Below Knee | 8 | 106.5 | 122.5 | 146.4 | 165.4 | 171.4 |
|  | Trail Above Knee | 3 | 86.2 | 102.6 | 152.0 | 167.1 | 172.2 |
|  | Trail Below Knee | 7 | 101.0 | 121.4 | 141.7 | 167.1 | 181.8 |
| 6-Iron | Lead Above Knee | 5 | 71.8 | 107.0 | 120.6 | 125.2 | 138.3 |
|  | Lead Below Knee | 8 | 87.3 | 97.6 | 118.1 | 131.8 | 133.0 |
|  | Trail Above Knee | 3 | 69.2 | 81.8 | 119.8 | 131.3 | 135.1 |
|  | Trail Below Knee | 7 | 72.2 | 87.2 | 111.1 | 125.3 | 137.0 |
| Pitching Wedge | Lead Above Knee | 5 | 51.7 | 80.9 | 93.3 | 97.8 | 109.5 |
|  | Lead Below Knee | 8 | 66.3 | 77.0 | 90.2 | 104.7 | 106.4 |
|  | Trail Above Knee | 3 | 52.1 | 63.9 | 99.0 | 106.8 | 109.4 |
|  | Trail Below Knee | 7 | 57.5 | 68.1 | 87.3 | 99.6 | 110.8 |

### Launch angle percentiles for Amputation Location and Side

|  | Amputation Location and Side | N | Launch Angle (deg) | | | | |
| --- | --- | --- | --- | --- | --- | --- | --- |
|  |  |  | 10th | 25th | 50th | 75th | 90th |
| Driver | Lead Above Knee | 5 | 6.0 | 9.4 | 11.3 | 11.6 | 11.8 |
|  | Lead Below Knee | 8 | 6.8 | 8.8 | 12.0 | 16.2 | 17.2 |
|  | Trail Above Knee | 3 | 10.0 | 10.9 | 13.8 | 14.3 | 14.4 |
|  | Trail Below Knee | 7 | 9.2 | 10.3 | 10.6 | 11.1 | 13.0 |
| 6-Iron | Lead Above Knee | 5 | 10.8 | 12.0 | 15.7 | 15.9 | 16.0 |
|  | Lead Below Knee | 8 | 7.7 | 10.4 | 14.1 | 16.0 | 16.3 |
|  | Trail Above Knee | 3 | 11.4 | 12.2 | 14.8 | 15.2 | 15.3 |
|  | Trail Below Knee | 7 | 9.6 | 14.9 | 17.2 | 17.8 | 22.0 |
| Pitching Wedge | Lead Above Knee | 5 | 20.4 | 23.9 | 27.0 | 29.1 | 34.2 |
|  | Lead Below Knee | 8 | 14.0 | 19.0 | 21.7 | 25.4 | 29.4 |
|  | Trail Above Knee | 3 | 24.5 | 24.5 | 24.6 | 27.5 | 28.5 |
|  | Trail Below Knee | 7 | 16.2 | 21.5 | 26.5 | 33.3 | 35.1 |

### Spin rate percentiles for Amputation Location and Side

|  | Amputation Location and Side | N | Spin Rate (rpm) | | | | |
| --- | --- | --- | --- | --- | --- | --- | --- |
|  |  |  | 10th | 25th | 50th | 75th | 90th |
| Driver | Lead Above Knee | 5 | 2017 | 2463 | 2833 | 3193 | 3380 |
|  | Lead Below Knee | 8 | 2093 | 2805 | 3030 | 3512 | 4270 |
|  | Trail Above Knee | 3 | 2171 | 2251 | 2491 | 4004 | 4509 |
|  | Trail Below Knee | 7 | 2629 | 2688 | 2742 | 3319 | 4063 |
| 6-Iron | Lead Above Knee | 5 | 4387 | 5474 | 5953 | 6560 | 7233 |
|  | Lead Below Knee | 8 | 4081 | 4410 | 5068 | 6090 | 7091 |
|  | Trail Above Knee | 3 | 4483 | 5024 | 6647 | 6758 | 6795 |
|  | Trail Below Knee | 7 | 4827 | 5102 | 5529 | 5738 | 5833 |
| Pitching Wedge | Lead Above Knee | 5 | 5254 | 5477 | 7107 | 8658 | 9830 |
|  | Lead Below Knee | 8 | 3728 | 3947 | 7167 | 8154 | 9360 |
|  | Trail Above Knee | 3 | 4784 | 5317 | 6918 | 7278 | 7398 |
|  | Trail Below Knee | 7 | 3850 | 4628 | 5076 | 7773 | 8831 |

### Spin axis tilt percentiles for Amputation Location and Side

|  | Amputation Location and Side | N | Spin Axis Tilt (deg) | | | | |
| --- | --- | --- | --- | --- | --- | --- | --- |
|  |  |  | 10th | 25th | 50th | 75th | 90th |
| Driver | Lead Above Knee | 5 | 0.3 | 2.8 | 5.3 | 17.0 | 31.3 |
|  | Lead Below Knee | 8 | -18.7 | -13.1 | 0.8 | 8.2 | 17.8 |
|  | Trail Above Knee | 3 | 1.1 | 1.5 | 2.5 | 11.7 | 14.8 |
|  | Trail Below Knee | 7 | -0.9 | -0.1 | 2.5 | 10.8 | 13.0 |
| 6-Iron | Lead Above Knee | 5 | -3.2 | 0.7 | 3.3 | 9.0 | 21.4 |
|  | Lead Below Knee | 8 | -10.7 | -8.9 | -1.6 | 5.1 | 9.6 |
|  | Trail Above Knee | 3 | 1.1 | 1.4 | 2.5 | 3.3 | 3.5 |
|  | Trail Below Knee | 7 | -8.9 | -3.5 | 3.5 | 11.2 | 20.1 |
| Pitching Wedge | Lead Above Knee | 5 | -1.4 | -0.4 | 2.6 | 7.1 | 15.8 |
|  | Lead Below Knee | 8 | -7.5 | -6.8 | -1.5 | 3.1 | 6.9 |
|  | Trail Above Knee | 3 | -4.9 | -3.8 | -0.3 | 4.7 | 6.4 |
|  | Trail Below Knee | 7 | -5.2 | -2.1 | -0.9 | 5.4 | 11.9 |

### Carry distance percentiles for Amputation Location and Side

|  | Amputation Location and Side | N | Carry Distance (yards) | | | | |
| --- | --- | --- | --- | --- | --- | --- | --- |
|  |  |  | 10th | 25th | 50th | 75th | 90th |
| Driver | Lead Above Knee | 5 | 67.2 | 191.7 | 239.2 | 250.0 | 253.8 |
|  | Lead Below Knee | 8 | 150.5 | 177.7 | 233.4 | 255.0 | 270.4 |
|  | Trail Above Knee | 3 | 95.1 | 132.4 | 244.5 | 272.1 | 281.3 |
|  | Trail Below Knee | 7 | 122.1 | 168.8 | 216.2 | 270.4 | 289.2 |
| 6-Iron | Lead Above Knee | 5 | 67.8 | 135.2 | 166.6 | 176.6 | 194.4 |
|  | Lead Below Knee | 8 | 96.0 | 121.3 | 167.6 | 185.9 | 191.8 |
|  | Trail Above Knee | 3 | 57.6 | 83.8 | 162.2 | 182.0 | 188.6 |
|  | Trail Below Knee | 7 | 78.0 | 109.5 | 155.7 | 176.7 | 184.1 |
| Pitching Wedge | Lead Above Knee | 5 | 42.7 | 98.1 | 116.6 | 126.4 | 137.7 |
|  | Lead Below Knee | 8 | 55.0 | 90.1 | 117.5 | 137.3 | 143.1 |
|  | Trail Above Knee | 3 | 47.2 | 66.0 | 122.2 | 141.5 | 147.9 |
|  | Trail Below Knee | 7 | 56.9 | 78.6 | 112.1 | 131.0 | 136.4 |

### Offline distance percentiles for Amputation Location and Side

|  | Amputation Location and Side | N | Offline Distance (yards) | | | | |
| --- | --- | --- | --- | --- | --- | --- | --- |
|  |  |  | 10th | 25th | 50th | 75th | 90th |
| Driver | Lead Above Knee | 5 | -14.1 | -5.1 | 5.3 | 11.7 | 15.8 |
|  | Lead Below Knee | 8 | -5.5 | -0.3 | 2.4 | 8.7 | 19.1 |
|  | Trail Above Knee | 3 | -6.7 | -3.7 | 5.2 | 10.1 | 11.7 |
|  | Trail Below Knee | 7 | -12.1 | -10.4 | 1.6 | 8.5 | 14.3 |
| 6-Iron | Lead Above Knee | 5 | -9.0 | -8.7 | 0.1 | 3.1 | 3.5 |
|  | Lead Below Knee | 8 | -4.5 | -3.4 | 0.5 | 4.8 | 8.0 |
|  | Trail Above Knee | 3 | -1.2 | -0.5 | 1.7 | 6.6 | 8.2 |
|  | Trail Below Knee | 7 | -14.3 | 0.8 | 3.5 | 9.0 | 13.3 |
| Pitching Wedge | Lead Above Knee | 5 | -6.2 | -4.6 | -1.0 | 0.0 | 1.5 |
|  | Lead Below Knee | 8 | -2.3 | -0.7 | 1.0 | 2.7 | 4.6 |
|  | Trail Above Knee | 3 | -1.4 | 0.0 | 4.1 | 4.8 | 5.0 |
|  | Trail Below Knee | 7 | -3.7 | -2.6 | -2.1 | 1.4 | 8.3 |
